# A Review on Adsorption of Fluoride from Aqueous Solution

**DOI:** 10.3390/ma7096317

**Published:** 2014-09-05

**Authors:** Mirna Habuda-Stanić, Maja Ergović Ravančić, Andrew Flanagan

**Affiliations:** 1 Department of Chemistry and Ecology, Faculty of Food Technology, Josip Juraj Strossmayer University of Osijek, Franje Kuhača 20, HR-31000 Osijek, Croatia; 2 Department of Food Technology, University of Applied Sciences in Požega, Vukovarska 17, HR-34000 Požega, Croatia; E-Mail: mergovic@vup.hr; 3 HSE Public Analyst’s Laboratory Galway, University College Hospital, Seamus Quirke Road, Galway, Ireland; E-Mail: andrew.flanagan@hse.ie

**Keywords:** adsorption, fluoride, adsorbents, fluoride removal, water

## Abstract

Fluoride is one of the anionic contaminants which is found in excess in surface or groundwater because of geochemical reactions or anthropogenic activities such as the disposal of industrial wastewaters. Among various methods used for defluoridation of water such as coagulation, precipitation, membrane processes, electrolytic treatment, ion-exchange, the adsorption process is widely used. It offers satisfactory results and seems to be a more attractive method for the removal of fluoride in terms of cost, simplicity of design and operation. Various conventional and non-conventional adsorbents have been assessed for the removal of fluoride from water. In this review, a list of various adsorbents (oxides and hydroxides, biosorbents, geomaterials, carbonaceous materials and industrial products and by-products) and its modifications from literature are surveyed and their adsorption capacities under various conditions are compared. The effect of other impurities on fluoride removal has also been discussed. This survey showed that various adsorbents, especially binary and trimetal oxides and hydroxides, have good potential for the fluoride removal from aquatic environments.

## 1. Introduction

Fluorine (F_2_) is a pale, yellow-green, corrosive gas which almost cannot be found in natural environment in elemental form due to its high electronegativity and reactivity. Fluoride (F^−^) is a fluorine anion characterized by small radius, great tendency to behave as ligand and easiness to form a great number of different organic and inorganic compounds in soil, rocks, air, plants and animals. Some of those compounds are quite soluble in water, so fluoride is present in surface and groundwater as an almost completely dissociated fluoride ion [1,2].

The presence of naturally occurring fluorides or added fluoridated salts in drinking water allows its easy entrance in the body via the gastrointestinal tract [3,4]. The epidemiological studies reveal that drinking water is the major source of fluoride daily intake and continuous consumption of drinking water with heightened fluoride concentrations (>1.5 mg/L) can induces birth, reproduction and immunological defects [5,6], dental and skeletal fluorosis [7,8,9,10,11,12,13,14]. Besides drinking water, fluoride can also enter into the human body through food, industrial exposure, drugs, cosmetics,* etc.* [1].

Fluoride occurrence and concentrations in water resources, surface water and groundwater, depends on several contributing factors, such as pH, total dissolved solids, alkalinity, hardness and geochemical composition of aquifers [1,15,16,17,18,19,20], but in many countries worldwide, elevated fluoride concentrations are result of fluorine polluted waste water discharges. Such waste waters are usually produced by the superphosphate fertilizer industry [21,22], glass and ceramic manufacturing processes [23,24], aluminum and zinc smelters [25,26,27], steel production, uranium enrichment facilities, coal fired power stations, beryllium extraction plants, oil refineries [26,28,29,30], photovoltaic solar cells industry [31,32], silicon based high tech-semiconductors production [33,34,35,36] and in municipal waste incineration plants through HF emissions caused by incinerating of fluorinated plastic, fluorinated textiles or CaF2 decomposition in waste sludge [37].

Due to all previously mentioned fluoride pollutions and health problems that it causes, the World Health Organization (WHO) has specified the tolerance limit of fluoride content of drinking water as 1.5 mg/L [38]. Various technologies, presented in Table 1, are currently available to remove fluoride from water, such as coagulation and precipitation [30,39,40,41,42,43], membrane processes [44,45,46,47,48,49,50,51,52], electrochemical treatments [26,53,54,55,56,57,58,59,60,61,62], ion-exchange and its modification [63,64,65,66,67,68,69], but the adsorption process is generally accepted as the cheapest and most effective method for removal fluoride from water [20,25,29,70,71,72,73].

**Table 1:** Comparison of fluoride removal technologies [20,29,40,52,54,74,75].

**Technology**	**Advantages**	**Disadvantages**
Coagulation/precipitation: *calcium hydroxide*; *aluminum hydroxide*	High efficiency; commercially available chemical	Expensive, efficiency depends of pH and presence of co-ions in water, adjustment and readjustment of pH is required, elevated residual aluminum concentration, formation of sludge with high amount of toxic aluminum fluoride complex and high amount of retained water (sludge dewatering is required prior disposal)
Membrane filtration: *reverse osmosis*; *nanofiltration*	High efficiency; remove other contaminates	High capital high running and maintenance costs toxic waste water produced
Electrochemical treatments: *dialysis*; *electro-dialysis*; *electro-coagulation*	High efficiency; high selectivity	High cost during installation and maintenance
Ion-exchange: *Strong basic anion-exchange resin with quaternary ammonium functional groups*	High efficiency	Expensive, vulnerable to interfering ions (sulfate, phosphate, chloride, bicarbonate,* etc.*), replacement of media after multiple regenerations, used media present toxic solid waste, regeneration creates toxic liquid waste, efficiency highly pH-dependent
Adsorptive materials: *activated alumina*; *activated carbons*; *other natural and synthetic adsorbents*	Greater accessibility, low cost, simple operation, availability of wide range of adsorbents	High efficiency often demand adjustment and readjustment of pH, some common water ions can interfere fluoride adsorption

## 2. Fluoride Remediation by Adsorption

Proponents of adsorption technology argue that the technique is economical efficient and produces high quality water. The removal of fluoride by adsorption methods has been widely studied in recent years and interest is growing in the use of high-valency metals to functionalized sorbents [76]. Adsorption of fluoride on to solid absorbant usually occurs through three phases [77,78]:

(1) diffusion or transport of fluoride ions to the external surface of the adsorbent from bulk solution across the boundary layer surrounding the adsorbent particle, called external mass transfer;
(2) adsorption of fluoride ions on to particle surfaces;
(3) the adsorbed fluoride ions probably exchange with the structural elements inside adsorbent particles depending on the chemistry of solids, or the adsorbed fluoride ions are transferred to the internal surfaces for porous materials (intra particle diffusion).

Adsorption depends on ions (adsorbate) in fluid diffusing to the surface of a solid (adsorbent), where they bond with the solid surface or are held there by weak intermolecular forces [79].

Adsorption studies pointed most important characteristics which determined adsorbent suitability for practical application: adsorption capacity, selectivity for fluoride ions, regenerability, compatibility, particle and pore size, and cost while fluoride removal efficiency always depends on raw water quality profile,* i.e.*, initial fluoride concentration, pH, temperature, contact time and adsorbent dosage [29,74,75,77,78,80].

Among the above-listed characteristics and process parameters, adsorbent’s selectivity for fluoride ions seem to be most important adsorbent characteristic since some of adsorbents showed high efficiency in test bench but, in the same time, fail under real conditions at water treatment plant due to reducing the effective adsorption capacity caused by adsorbents’ active sites occupation by other co-ions present in treated water. Defined as the ratio of the capacity of one component to that of another at a given fluorine concentration, selectivity generally approaches a constant value as concentration drops towards zero. So, the main task of scientists and experts is to find or develop cheap, efficient and environment-friendly, but highly selective adsorbent with high effective adsorption capacity [29,75,80,81,82].

A wide variety of adsorbents and their modifications have been tested for the removal of fluoride from water. These include activated carbons [81,82,83,84,85,86], activated alumina [87,88,89,90], bauxite [89,91,92,93,94,95,96,97,98,99,100,101,102], hematite [95,103,104,105], polymeric resins [67,96,97,106], activated rice husk [83,98,99,107], brick powder [100], pumice stone [101,108,109], red soil, charcoal, brick, fly ash, serpentine [102,110,111], seed extracts of *Moringaoleifera* [98], granular ceramics [112], chitin, chitosan and alginate [95,113,114,115,116,117,118,119], modified ferric oxide/hydroxide [120,121,122,123,124,125,126], hydroxyapatite (HAP) [106,107,127,128,129], zirconium and cerium modified materials [77,130,131,132,133,134,135,136,137,138,139,140], titanium-derived adsorbent [141,142,143], schwertmannite [144], modified cellulose [145,146], clays [147,148,149,150,151], zeolite [74,76,152,153,154,155,156,157] and magnesium-modified sorbent [128,140,158]. Among all the above listed adsorbents, the best results and higher adsorption capacities are shown by different metal oxides and hydroxides, especially those prepared in nano-form.

However, with fluoride concentration decreasing, a lot of adsorbents lose the fluoride removal capacity, the lowest limit for fluoride reduction by most of the adsorbents is 2 mg/L; therefore, they are not suitable for drinking water, especially as some of them can only work at an extreme pH value, such as activated carbon which is only effective for fluoride removal at pH < 3.0 [159].

## 3. Adsorbents for Fluoride Removal

### 3.1. Oxides and Hydroxides

Many researchers reported successfully fluoride remediation using different metal oxides and hydroxides characterized by high surface area and numerous of them have used iron oxide as an adsorbent to treat heavy metals, anions, and hazardous elements in wastewater [160]. Hydrous titanium dioxide (TiO_2_) has been found to be a potential selective adsorbent for fluoride ions, as well as halogens and arsenic compounds. Ishihara* et al.* [161] demonstrated the potential for selective adsorption of fluoride ions, and the characteristics of the adsorption-desorption cycle with titanium tetrahydroxide dry powder, Ti(OH)_4_. To apply the titanium hydroxide to highly effective equipment by loading column, mesoporous materials, brushes,* etc.*, in order to adsorb the fluoride ion, the gel-like titanium hydroxide-derived adsorbents from titanium oxysulfate, TiO(SO_4_) was prepared. It was confirmed that the gel-like adsorbent had as high an adsorption ability as the Ti(OH)_4_ powder. The adsorbent had high adsorption abilities for fluoride ions, even at low fluoride concentrations and had selectivity for fluoride ions with coexisting chloride, nitrate and sulfate ions. The adsorbent could remove fluoride ions in real wastewater to below 0.8 mg/L. The adsorbent could desorb fluoride ions by controlling the pH of the solution to the alkaline region, and the cycle stability of fluoride ion adsorption in the adsorbent is sufficiently high for recovery of fluoride ions. The Langmuir and Freundlich adsorption isotherm models were applied to equilibrium data at pH = 3. The equilibrium data fitted the Langmuir and Freundlich isotherms very well [141].

Titanium hydroxide-derived adsorbents, synthesized in a titanium hydroxide gel form, showed high adsorption abilities for fluoride ions, and its good adsorption characteristics and selectivity were noted even in the presence of coexisting chloride, nitrate and sulfate ions. The adsorbent could remove fluoride ions below 0.8 mg/L from solution with an initial fluoride concentration of 50 mg/L [141].

Chen* et al.* [142] investigated the possibility of fluoride removal using a nano-adsorbent bimetallic oxide adsorbent synthesized by co-precipitation of Fe(II) and Ti(IV) sulfate solution using ammonia titration at room temperature. The influences of the washing and drying methods, Fe/Ti molar ratio, and calcination temperature used in the preparation on the morphology, crystallization, surface structure and adsorption capacity were investigated. Experimental results show that a Fe–Ti bimetallic oxide adsorbent had a Langmuir adsorption capacity of 47.0 mg/g, which was much higher than the adsorption capacities reported for a pure Fe oxide or Ti oxide adsorbent. Authors explained this as being a synergistic interaction between Fe and Ti in Fe–O–Ti bonds on the adsorbent surface and hydroxyl groups which provided the active sites and formation of Fe–O–Ti–F bonds and economical fluoride removal from drinking water.

Iron and aluminum binary oxide (FeAlO*_x_*H*_y_*), aluminum oxyhydroxide (AlO*_x_*H*_y_*) and iron oxyhydroxide (FeO*_x_*H*_y_*) were investigated with the aim to evaluate their removal efficacy towards arsenate and fluoride, to determine the effects of pH and the ratios of iron to aluminum on the removal of arsenate and fluoride, and finally, to investigate the competitive adsorption between arsenate and fluoride onto FeAlO*_x_*H*_y_* [122]. Batch adsorption experiments were conducted at 25 °C using arsenate and fluoride solutions with initial concentrations of 0.2 mM and initial pH between 4 and 9. FeO*_x_*H*_y_* shows a high removal capability towards arsenate but exhibits little efficacy to fluoride removal in systems where both arsenate and fluoride co-exist or for solutions of fluoride alone. AlO*_x_*H*_y_* shows good efficiency when simultaneously arsenate and fluoride were removed within a wide pH range (4–11) and best results were obtained at the weakly acidic pH = 6 because of the effect of electrostatic force at different pH. Generally, authors observed better arsenic removal (up to 94.8%) with used adsorbents. Up to 18.4% of fluoride initial concentration was removed when FeO_x_H*_y_* was used, up to 29.4% fluoride was removed when AlO*_x_*H*_y_* was used, and usage of FeAlO*_x_*H*_y_* removed up to 64.5% of fluoride.

Chai* et al.* [121] applied sulfate-doped Fe_3_O_4_/Al_2_O_3_ nanoparticles with magnetic separability for fluoride removal from drinking water. Adsorption experiments were performed with 100 mL fluoride solution with initial fluoride concentrations ranging from 2 mg/L to 160 mg/L and 0.1 g of adsorbent during 7 h. Adsorption experiments was conducted at 25 °C and initial pH value of used fluoride solutions were from 2 to 12. Authors of this study also investigated the effects of competing anions (chloride, nitrate, sulfate and phosphate) on fluoride adsorption under a fixed initial competing anions concentration (2 mmol/L), and initial fluoride concentrations of 5 mg/L and 20 mg/L at pH = 7.0. Due to the Langmuir model, the calculated adsorption capacity for fluoride of the sulfate-doped Fe_3_O_4_/Al_2_O_3_ was 70.4 mg/g at pH = 7.0. The optimum pH range for fluoride removal was from 4.0 to 10.0 which makes the tested adsorbent suitable for applicability in natural water treatments. Authors emphasized that ion exchange of sulfate by fluoride and formations of inner-sphere fluoride complex were the important mechanisms for fluoride removal by used adsorption material, and that competing anions, except phosphate, did not inhibit fluoride removal which suggest that used nanoadsorbent had a high selectivity for fluoride.

Magnesium-doped nano ferrihydrite was used for fluoride removal by Mohapatra* et al.* [162]. Study evaluates synthesized Mg-doped nano ferrihydrite obtained by varying Mg content in the range of 0.39%–1.12% since their preliminary test showed that increase of Mg content on nano ferrihydrite from 0.39% to 0.98% increased fluoride removal from 66% to 91%. Batch adsorption experiments were carried out by varying contact time (30–480 min), initial pH (1.0–10), initial fluoride concentration (10–150 mg/L), adsorbent dose (0.5–4 g/L) and temperature (20–45 °C). In this study, the authors also investigated the effects of competing chloride and sulfate anions (up to 50 mg/L). The highest fluoride removal and adsorption capacity of 64 mg/g was observed when 0.98% Mg-doped ferrihydrite was used and time data fitted well to pseudo second order kinetic model. Characterization of used Mg-doped ferrihydrite by X-ray diffraction (XRD), transmission electron microscopy, selected area electron diffraction and thermo-gravimetric (TG) and differential thermal analyses showed that after fluoride adsorption, the particles were more dispersed having better crystallinity and the presence of fluoride on the loaded adsorbent was confirmed. A fluoride desorption test showed that under different pH and contact time about 89% fluoride could be desorbed.

García-Sánchez* et al.* [123] examined fluoride removal using aluminum modified iron oxides in a fixed bed column experiments performed (borosilicate glass column of 9 mm). The column studies were conducted to evaluate the effect of different bed weights on the breakthrough curves. Fixed bed experiments were carried out using a solution with initial fluoride concentration of 4 mg/L (pH = 6.4) and drinking water (pH = 7.4), flow rate of 1 mL/min, 2, 4 and 6 g of aluminum modified iron oxides with bed depths of 3.5, 7.0 and 10.5 cm, respectively. The obtained results show that the throughput volume of used fluoride solutions or drinking water, increases with increasing bed height, due to the availability of more number of sorption sites due to increase of the total surface area. Measurements show that average pH values of eluted fluoride solutions were 7.2 for model solution and 7.3 for drinking water. Finally, authors reported that highest fluoride adsorption capacities at the breakthroughs were 0.509 mg/g (bed depth 3.5 cm; bed weight 2 g) after 254.3 min when fluoride solution was passed through the column and 0.296 mg/g (same bed depth and bed weight as previous) after 148 min when drinking water was passed through the column.

The performance and mechanism of calcined Mg/Fe layered double hydroxides, synthesized by co-precipitation method, was investigated for simultaneous fluoride and arsenate removal from aqueous solution by Kang* et al.* [124]. Adsorption experiments were performed with the aim of determining the effect of various factors on fluoride removal efficiency such as material preparation, effect of Mg/Fe molar ratio and calcination temperature, while adsorption isotherms were carried out to predict adsorption mechanism, and to determine the optimum conditions for the maximum fluoride and arsenate removal by calcined Mg/Fe layered double hydroxides. Study results showed that the highest fluoride removal was obtained when Mg/Fe layered double hydroxides calcined at 400 °C, while due to used Langmuir isotherm model, maximum adsorption capacities of fluoride was 50.91 mg/g. Authors also emphasized that adsorption mechanism for fluoride involved surface adsorption, ion exchange interaction and the original layered double hydroxide structure reconstruction by intercalation of fluoride and arsenate ions into interlayer region.

Poursaberi* et al.* [132] synthesized adsorbent for fluoride removal using Fe_3_O_4_ nanoparticles, obtained by co-precipitation of ferrous and ferric ions in a 3-aminopropyl triethoxysilane and additionally functionalized them using a zirconium(IV) porphyrin complex. Batch experiments using 10 mL of fluoride solution with initial fluoride concentration of 10 mg/L, and various amounts of adsorbent (3–500 mg) showed high selectivity of used adsorbent regarding fluoride extraction. Under optimal conditions (contact time 20 min, pH = 5.5 and adsorbent dosage of 100 mg), up to 92% of fluoride was removed from initial solution with 10 mg/L. Experiments regarding adsorbent efficiency due to presence of coexisting anions (sulfate, nitrate, nitrite, bromide and chloride) in different molar ratios (from 1:1 to 1:100) showed that the removal percentage of fluoride remained within the tolerance limit in the presence of a 10-fold concentration of the chloride and bromide, 50-fold of sulfate and 100-fold of nitrite and nitrate concentrations. An adsorption test was conducted with real water contamined with fluoride (average fluoride concentration of 35.5 mg/L). It showed maximal fluoride removal of 92% (residual fluoride concentration of 2.8 mg/L). In a conclusion of this study, the authors emphasized that advantage of synthesized zirconium(IV)-metalloporphyrin grafted Fe_3_O_4_ nanoparticles are the ease of separation by an external magnetic field, possibility of simple recovery after washing with a basic aqueous solution and its reuse for up to five cycles.

Using the extrusion method, a granular zirconium-iron oxide composed of amorphous and nano-scale oxide particles was prepared and its adsorption characteristic due to fluoride removal were investigated by Dou* et al.* [126]. Fluoride removal was systematically evaluated under various operating conditions: initial fluoride concentration (10–150 mg/L), pH value (3–11), reaction time and co-existing substances using fluoride solution and real fluoride-containing groundwater samples via batch and column tests. The leachability potential of the used granular zirconium-iron oxide was tested using the toxicity characteristic leaching procedure. Due to the results of batch tests, the authors reported a high adsorption capacity of 9.80 mg/g under an equilibrium concentration of 10 mg/L and at pH = 7.0, and efficient fluoride removal over a wide pH range (3.5–8.0), especially between pH 6.0 and 8.0. Fluoride adsorption onto granular zirconium-iron oxide followed pseudo-second-order kinetics and could be described by the Freundlich equilibrium model. The co-existing substances, considering their real concentrations in natural groundwater such chloride, sulfate, nitrate, silicate, phosphate, humic acids,* etc.*, did not evidently inhibit fluoride removal with the exception of bicarbonate which authors refers as evidence of high selectivity of used adsorbent for fluoride. Dou* et al.* [130] studied the efficiency of synthesized hydrous zirconium oxide using batch adsorption tests while fluoride removal mechanism was investigated by surface titration, electrophoretic measurement, spectroscopic techniques and surface complexation models. Batch isotherm experiments were carried out using solutions with initial fluoride concentrations between 2 mg/L and 120 mg/L, adsorbent dose of 0.3 g/L, pH were kept at 7.0 and 4.0 at 25 °C during 24 h. Kinetics experiments were performed at room temperature using solutions with initial fluoride concentrations of 20 mg/L and 100 mg/L with adsorbent dose of 0.3 g/L at pH = 7.0. Authors also examined effect of pH and co-existing anions on fluoride removal. Results confirmed that hydrous zirconium oxides have fluoride adsorption capacities of 124 and 68 mg/g at pH = 4 and 7, respectively. Kinetic studies showed that fluoride adsorption followed a pseudo-second-order rate law. Surface titration, electrophoretic measurement, spectroscopic techniques and surface complexation models showed that fluoride removal by hydrous zirconium oxides occurred by the exchange of surface hydroxyl groups with fluoride, and by the electrostatic interaction between charging surface and fluoride. No surface precipitation of NaF or crystalline ZrF_4_ was observed at the surface of hydrous zirconium oxide. Authors also reported that capacity of synthesized adsorbent drops as pH increases due to the changes of pH-dependent electrostatic force existing between the sorbent surface and fluoride. A slight effect of silicate nitrate, chloride, and sulfate, on fluoride removal was observed at all concentrations, while strong inhibition of fluoride removal was noted when phosphate, arsenate and bicarbonate were present at a concentration of 100 mg/L.

Koilraj and Kannan [134] used nitrate containing zirconium/chromium layered double hydroxides with Zn/Cr atomic ratio of 2.0, 3.0 and 4.0 for fluoride removal from water. The fluoride uptake studies were conducted in batch mode by using 1 g/L of adsorbent material and different initial fluoride concentration (0–100 mg/L) solution stirred magnetically at 600 rpm for 3 h. Obtained results show maximum fluoride uptake capacity of 31 mg/g for synthesized adsorbent and uptakes were increased with an increase in layered double hydroxides loading. Fluoride uptake studies in presence of competing anions (carbonate, sulfate, phosphate, nitrate,* etc.*) revealed a preferential uptake of certain monovalent anions and divalent anions. Practical usage of adsorbents was examined via column tests using polysulfone/zirconium-chromium layered double hydroxides with varying the amount of layered double hydroxides. Results show that usage of composite material inferred better aqueous diffusivity than powered layered double hydroxides suggesting that polysulfone could be used as a suitable column material. The uptake capacities calculated based on the time, flow rate and concentration up to the breakthrough points were 1.0, 1.7, 6.9 mg/g while the total uptake capacities were 1.8, 4.9 and 12.5 mg/g for composite materials containing 9%, 17% and 38% of layered double hydroxides, respectively. Column study for the uptake of fluoride from real water (fluoride concentration of 12.6 mg/L) revealed that, at the initial stage (<20 min), all anions were taken up by the used adsorbent implying poor selectivity when real water samples were treated.

Kinetics, equilibrium and thermodynamic aspects of fluoride removal from drinking water using meso-structured zirconium phosphate examined Swain* et al.* [131]. Batch experiments were carried out to investigate the effects of adsorbent dose, pH, contact time, initial fluoride concentration and presence of other ions (chloride, sulfate, nitrate, bicarbonate and phosphate) on fluoride removal. The effects of co-anions such as chloride, sulfate, nitrate, bicarbonate, phosphate upon the adsorption process were also studied. Maximum fluoride uptake was obtained at pH = 6.0. The adsorption process was studied using different adsorption isotherm models which suggested that adsorption probably proceeded by an ion-exchange mechanism. Study results show that the percentage of fluoride removal increases with increasing of adsorbent dose and nearly 96% of fluoride was removed with a dose of 3.0 g/L. Presence of other ions showed no stronger effect upon fluoride uptake. A leaching study provided with NaOH showed good results and, therefore, adsorbent can sustainably be utilized for a number of cycles.

A similar group of authors synthesized, characterized and examined fluoride adsorption using environmental friendly sol-gel zirconium(IV)-ethylenediamine hybrid sorbent [133]. Fluoride removal was tested using variation of solution parameters. Authors reported fluoride removal over 99% when 0.1 g of zirconium(IV)-ethylenediamine hybrid sorbent was used with 100 ml of fluoride solution (initial fluoride concentration 10 mg/L, pH = 7.0) during 60 min of adsorption. The adsorption process was followed by a pseudo second order kinetics with theoretical adsorption capacity and experimental adsorption capacity being close to each other. Desorption test showed that adsorbed fluoride can easily be desorbed from the adsorption material by 0.1 M NaOH. The tested effect of the presence of co-anions (nitrate, chloride, sulfate) showed no significant impact on fluoride removal.

Fluoride removal efficiency of CeO_2_-ZrO_2_ nanocages prepared by Kirkendall effect and a study of its structure was conducted by Wang* et al.* [135]. Due to results of batch adsorption study, the porous CeO_2_-ZrO_2_ nanocages showed the maximum capacity was calculated to be 175 mg/g at pH = 4.0 and isotherm are described well by the Langmuir model. Testing the effect of co-existent ions showed that chloride and arsenate in high concentrations have adverse effects on fluoride adsorption, while the presence of sulfate has no influence on the fluoride adsorption. Based on the electrophoretic measurement, spectroscopic techniques and surface complexation models, authors emphasized that adsorption mechanism of the adsorbent for fluoride probably could involve anion exchange and electrostatic interaction. Testing the efficiency of synthetized CeO_2_-ZrO_2_ nanocages with a groundwater sample naturally containing high fluoride concentration (2.820 mg/L) using adsorbent dose of 0.4 g/L, the residual fluoride concentration of 1.39 mg/L was achieved after one hour of treatment.

Srivastav* et al.* [163] used three types of hydrous bismuth oxides as fluoride adsorbents and reported the results of kinetics and equilibrium experiments and characterization analyses. The HBO_1_, HBO_2_ and HBO_3_ were synthesized varying the proportions of NaOH in the Bi_2_O_3_-HCl solution. Batch experiments were performed with following parameters: pH of the solutions was in the range of 4–12, adsorbent dosage was 50 g/L, initial fluoride concentrations were from 10 mg/L to 35 mg/L, concentration of competitive anions, while contact time were from 60 min to 360 min. Adsorbents HBO_1_, HBO_2_ and HBO_3_ were characterized using X-ray diffractometer, Fourier transform infrared spectroscopy (FTIR), scanning electron microscope (SEM) and Brunauer-Emmett-Teller (BET) surface area analyzer. HBO_1_ (lowest amount of NaOH) was observed to have highest fluoride removal with adsorption potentials increased from 0.064 mg/g to 0.196 mg/g at 20 mg/L of initial fluoride concentration, but reduced to 0.168 mg/g at 25 mg/L, and around 65% of initial fluoride concentration was reduced from solutions with initial concentration 5 mg/L and 10 mg/L. Characterization of the effectiveness of HBO_1_ has shown that it has a crystalline nature and the relevant peaks correspond to bismuth hydroxide and bismuth oxychloride in the material, while the FTIR analysis indicates presence of Bi–O and OH groups on the surface of HBO_1_. The SEM image reveals a rough surface and cotton like spongy structure. The author also noted that fluoride adsorption process onto used adsorbents followed a pseudo-second-order kinetic model better than the pseudo-first-order model and that the Langmuir isotherm appears to fit more closely than the Freundlich isotherm. Testing the effect of competitive anions revealed that the sulfate and chloride affect HBO_1_ was stronger than bicarbonate.

Babaeivelni and Khodadoust [143] examined adsorption of fluoride from water onto crystalline TiO_2_ powder composed mostly of anatase with a specific surface area of 56 m^2^/g. The aim of the adsorption experiments was to determine optimal adsorbent dosage, effect of initial fluoride concentration, pH, contact time, ionic strength and co-existing ions on the uptake of fluoride. Therefore, fluoride aqueous solution with initial pH value ranging between 2 and 11, different dosages of adsorbent (0.01–2 g) and initial fluoride concentration of 5 mg/L to 20 mg/L were used. Adsorption kinetics data showed that maximum adsorption of fluoride occurred within 3 h, following a pseudo-second order kinetics model. Adsorption isotherm data followed the Langmuir equation, indicating favorable adsorption of fluoride onto TiO_2_, while results from the Dubinin-Radushkevich model are indicative of physical adsorption of fluoride. Maximum adsorption of fluoride occurred within the pH range of 2–5, while approximately 75% of maximum adsorption was obtained in the pH range of 7–8 with rapidly declining adsorption when pH > 9. Higher bicarbonate concentrations,* i.e.*, higher alkalinity of solution, decrease in adsorption of fluoride onto the surface of TiO_2_.

The synthesized Fe–Al–Ce trimetal hydroxide adsorbent (Fe–Al–Ce) obtained by spraying of a Fe–Al–Ce nano-adsorbent suspension onto glass beads with acrylic-styrene copolymer latex used as a binder was used for fluoride removal from drinking water by Chen* et al.* [136]. The effects of coating temperature, latex/Fe–Al–Ce ratio and coating amount on granule compressive strength and adsorption capacity were investigated. Authors reported that due to fitted Langmuir isotherm model, the adsorption capacity of the Fe–Al–Ce adsorbent granules was calculated to be 0.37 mg/g. The increased strength of beads but decrease of adsorption capacity was also reported regarding the increase of coating temperature during adsorbent synthesis and increase of latex/Fe–Al–Ce ratio. Highest efficiency and maximum fluoride removal of 2.77 mg/g of used Fe–Al–Ce trimetal hydroxide adsorbent, was obtained when the granules was coated at 65 °C using a latex/Fe–Al–Ce ratio of 0.5:1. Specified fluoride adsorption capacity was noted when initial fluoride concentration was 0.001 M with initial pH = 7 used. Similar adsorbent was synthesized and examined in another study conducted by Wu* et al.* [125]. They used nano-adsorbents of Fe–Al–Ce trimetal hydroxide immobilized in porous polyvinyl alcohol in a form of composite granules (3–5 mm) for fluoride removal. Fluoride adsorption test conducted with 2 g/L adsorbent dose and 100 mL fluoride solution was 100 mL showed the adsorption capacity of 4.46 mg/g at an initial fluoride concentration of 19 mg/L and pH = 6.5.

Zhao* et al.* [137] examined granulated iron–aluminum–cerium hydroxide (Fe–Al–Ce) obtained using extrusion with cross-linked poly vinyl alcohol as the binder. Efficiency of synthesized trimetal absorbent was tested using fluoride solutions with different initial concentrations (10–250 mg/L) at pH = 7.0, column test with fluoride-spiked tap water (average fluoride concentration 5.0 m/L, average pH = 7.8) and groundwater with naturally elevated fluoride concentration (average fluoride concentration 3.7 mg/L, average pH = 8.2). Regeneration experiments were also conduced using NaAlO_2_ solution.

The granulated Fe–Al–Ce hydroxide exhibited a Langmuir maximum adsorption capacity of 51.3 mg/g at pH = 7.0. At the breakthrough point (fluoride residual concentration 1.0 mg/L), column tests showed fluoride adsorption capacity of 5.7 mg/g when fluoride-spiked tap water was treated and 3.2 mg/g when natural groundwater with heightened fluoride concentration was used. Structural analysis of synthesized adsorbent showed that fluoride was distributed evenly in the cross-section of the used Fe–Al–Ce hydroxide suggesting that most active sites inside the adsorbent were available for fluoride removal. A regeneration tests conducted with NaAlO_2_, showed good adsorbent performance and fluoride retention over 60% even after four adsorption-regeneration cycles.

Fluoride removal efficiency of synthesized Mg–Cr–Cl layered double hydroxide examined using batch test Mandal* et al.* [139] regarding adsorbent dosages, contact time, pH and initial fluoride concentration. Structural characteristics were investigated by X-ray powder diffraction, FTIR, TG analysis, differential thermal analysis, SEM. Results showed that 88.5% fluoride was removed at pH = 7 with an adsorbent dose of 0.6 g/100 mL solution and 77.4% at the same pH but with 10 mg/L of fluoride initial concentration and 100 mg/L of adsorbent dosage. Adsorption experiment data were fitted well with the Langmuir isotherm while measured thermodynamic constants showed that the adsorption process was spontaneous and endothermic in nature. Investigations into the effect of pH showed that adsorbent efficiency slowly decreased with increasing pH. Because of a lack of synthesized Mg–Cr–Cl layered double hydroxide, authors emphasized the impossibility of adsorbent regeneration.

Activated alumina showed good adsorption characteristics during fluoride removal from natural water [164]. Most of the available materials for defluoridation are expensive and technically non-feasible for rural areas. Hence, the need to find locally available defluoridation media for safe and easy use at both household and small community levels is desirable.

Activated alumina is known to be a very good adsorbent due to its high surface area, crystalline form, and activation process [70], but usually it works effectively at pH < 6 [165,166]. Stewart* et al.* [167] reported that fluoride sorption to alumina adsorbents is a complex process in which differences in surface morphology, pH, temperature, fluoride concentration and the presence of other major ions such as sulfates and bicarbonates interact to produce a wide range of reported specific sorption values. The adsorption of fluoride by activated alumina was found to vary over the entire solution pH, possibly because of electrostatic interactions between the surface of alumina and the dominant fluoride species in solution. It is feasible to achieve relatively high removals at solution pH between 4.0 and 6.0. The equilibrium model based on coordination chemistry described here can adequately describe the equilibrium behavior of the investigated systems. A second-ordered kinetic model based on surface-reaction fits the temporal adsorption data with fairly good accuracy. The effect of solution temperature was found to impose minimum impact on the adsorption process. The presence of a sulfate ion also inhibited the adsorption of fluoride ion to a certain extent by forming various aluminum-sulfate complexes in aqueous solutions. The experimental results indicated that the removal efficiency was influenced significantly by solution pH and the optimum operating pH was found to be in the range of 5–7. For neutral and acidic solutions, the adsorption capacities of fluoride by alumina were interfered with by the presence of sulfate [168]. Alumina possesses an appreciable defluoridation capacity of 1566 mg/kg. In order to improve its defluoridation capacity, it is aimed to prepare alumina polymeric composites using chitosan. Alumina/chitosan composite was prepared by incorporating alumina particles into the chitosan polymeric matrix, which can be made into any desired form *viz.*, beads, candles and membranes. The alumina/chitosan composite displayed a maximum defluoridation capacity of 3809 mg/kg* versus* alumina and chitosan alone (52 mg/kg). Alumina/chitosan composite possessed higher defluoridation capacity than alumina and chitosan. The defluoridation capacity of alumina/chitosan composite was not influenced by the pH of the medium and decreased in presence of bicarbonate. The sorption of fluoride on alumina/chitosan composite material followed the Freundlich isotherm. The nature of the reaction was spontaneous and endothermic. The fluoride removal of alumina/chitosan composite is mainly controlled by electrostatic adsorption and complexation mechanism. Field trial studies indicated that alumina/chitosan composite could be used as an effective defluoridating agent [169].

Layered double hydroxides, calcined at different temperatures have been demonstrated to recover their original layered structure in the presence of appropriate anions. In light of this so-called “memory effect”, a study of removal of fluoride from aqueous solution by calcined Mg–Al–CO_3_ layered double hydroxides has been carried out by Lv* et al.* [170]. The layered double hydroxides that were calcined at 500 °C had the highest capacity of removal of fluoride ion because of retention of intrinsic structure. The calcined layered double hydroxides with an Mg/Al ratio of 2 have a remarkable ability to adsorb anions. The adsorption loading is higher for the calcined Mg–Al-layered double hydroxides than for calcined Zn–Al and Ni–Al layered double hydroxides. Varying the conditions for fluoride removal, such as pH (5–10), initial fluoride concentration (10–200 mg/L), adsorbent dosage (1–4 g/1.8 L), temperature (30–80 °C) and influence of co-existing anions have been investigated. The influence of co-existing anions in fluoride aqueous solution indicates that the percentage of removal of fluoride increased in order phosphate < chloride ≈ sulfate < bromated << nitrate. It was found that maximum removal of fluoride from aqueous solutions was obtained in 6 h at pH = 6.0 with an initial concentration of 50 mg/L, and that the retention of fluoride ions by the calcined layered double hydroxides material was 98% or higher. The residual fluoride concentration was found to be 0.4 mg/L from an initial concentration of 20 mg/L, which meets the national standard for drinking water quality. The Freundlich isotherm and Langmuir isotherm were used to fit the data of equilibrium experiments. The results of XRD, FTIR and thermogravimetric analysis-mass spectrometry (TG-MS) demonstrate that the adsorption phenomenon is accompanied by rehydration with concomitant uptake of fluoride ions to rebuild the initial layered structure.

Fluoride adsorption onto granular ferric hydroxide was investigated using batch methods, under various ionic strength, pH, surface loading, and major co-existing anion conditions. Adsorption of fluoride on granular ferric hydroxide included an initial fast adsorption phase followed by a slow adsorption phase. Within the pH range of 2–11, fluoride adsorption equilibrium was not affected by ionic strength, but was significantly affected by pH. Maximum adsorption was achieved in the pH range of 3–6.5. Under the same pH condition, fluoride adsorption followed the Freundlich isotherm. Granular ferric hydroxide,* i.e.*, FeO(OH), is an effective and environmentally friendly sorbent for the removal of many toxic anions including arsenic, vanadium, and fluoride [171].

Wu* et al.* [172] developed a trimetal oxide as a fluoride adsorbent by coprecipitation of Fe(II), Al(III) and Ce(IV) salt solutions with a molar ratio of 1:4:1 under alkaline condition. A high adsorption capacity of 178 mg/g was acquired at pH = 7.0, which was highly competitive compared to other reported adsorbents. Fluoride adsorption was only partially inhibited by high concentrations of phosphate or arsenate but not affected by the presence of chloride and sulfate, and influenced by nitrate when NO_3_^−^-N concentration exceeded 50 mg/L.

Magnesium oxide or magnesia (MgO) is a well-known adsorbent showing extremely high defluoridation capacity. In order to overcome the limitations of MgO for field applications, Sairam Sundaram* et al.* [173] modify MgO with the abundant biomaterial chitosan to form an MgO /chitosan composite in a usable form. Fluoride removal from aqueous solution with MgO and MgO/chitosan composite was studied with batch equilibrium experiments. At equilibrium, MgO/chitosan composite has a defluoridation capacity of 4440 mg/kg, while MgO showed over two-times less capacity (2175 mg/kg). The defluoridation capacity of tested adsorbents was not influenced by pH and the presence of co-anions except bicarbonate ion. The adsorption process was followed the Freundlich isotherm.

Sasaki* et al.* [158] emphasized that problem of Mg-bearing and Mg-based sorbent, particularly the Mg–Al–CO_3_ layered double hydroxides, is in calcination that could result with the segregation of one metal and decrease of surface reactivity. Therefore, they repeatedly measured the sorption of fluoride on MgO-rich phases and calcined solid residues to evaluate their sorption density for fluoride and the stability of the chemically regenerated adsorbents. Five designed forms of MgO-phase were obtained (MgO-I, MgO-II, MgO-III, MgO-IV and MgO-V) and 0.1 g of each calcined product and fluoride model solution with initial concentration ranging from 1.24 mmol/L to 45.77 mmol/L (pH = 6.09) were used to evaluate the sorption density of fluoride and sorption isotherms at 25 °C. The sorption density of fluoride on the calcined product was the greatest for MgO-I and the smallest for MgO-II, although repeated calcinations did not show a clear trend in either the values of specific surface areas for the calcined products or the sorption efficiency of fluoride per unit mass and surface area. Authors suggested that differences in adsorption characteristic of used MgO-based adsorbents are probably due to increasing of the crystal size observed by transmission electron microscopy.

Investigating the behavior of fluoride in the water-kaolinite-MgO system with aim to evaluate the use of commercial-grade MgO as a fluoride immobilization agent, Suzuki* et al.* [151] reported that fluoride adsorption on kaolinite is strongly pH-dependent and better results were obtained at lower pH. When MgO was dosed (dosages ranging from 25 mg to 400 mg in 20 mL of fluoride solution with initial concentration of 100 mg/L), authors observed a strong increase in the aqueous pH,* i.e.*, adding 10 mg of MgO increased pH from 7.2 to 11.5 due to dissolution of MgO and releases of hydroxyl ions followed by the precipitation of Mg(OH)_2_.

Zhang* et al.* [140] investigated fluoride removal efficiency using non-thermal plasma modified CeO_2_/Mg–Fe layered double hydroxides. Experimental results indicated that the adsorption capacity was enhanced with non-thermal plasma surface modification of CeO_2_/Mg–Fe layered double hydroxides and authors reported the maximum adsorption capacity by Langmuir model of 60.4 mg/g, while the kinetic data of adsorption well fitted the pseudo-second-order model.

Some oxides and hydroxides, or their combination, poses magnetic properties what makes them easily removable from solutions. Nano-sized superparamagnetic zirconia material (ZrO_2_/SiO_2_/Fe_3_O_4_,) was applied for the sorption of fluoride from water and simulated industrial wastewater [174]. The experiments showed that superparamagnetic zirconia material possesses a satisfactory chemical stability at moderate acidic and basic conditions. Sorption and desorption develop very fast so that the equilibration is attained in few minutes. Fluoride sorption capacity increases with decreasing pH value. At pH = 4, the monolayer sorption capacity amounts to 14.7 mg/g (0.78 mmol/g). Experiments with simulated wastewater of the semiconductor industry demonstrated the selective elimination of fluoride, while effective regeneration was achieved using NaOH.

Zhao* et al.* [175] investigated fluoride removal from aqueous solution using synthesized Fe_3_O_4_-Al(OH)_3_ magnetic nanoparticles combining the advantages of hydrous aluminum oxide with magnetic separability and high affinity toward fluoride. The maximum adsorption capacity calculated by Langmuir equation was 88.48 mg/g at pH = 6.5. The main factors affecting the removal of fluoride, such as solution pH (5–9), temperature (25–50 °C), adsorption time (0–240 min), initial fluoride concentration (0–110 mg/L) and co-existing anions were investigated. The adsorption capacity increased with temperature and the kinetics followed a pseudo-second-order rate equation.

Due to the stability at low pH and its magnetic properties, schwertmannite was investigate for fluoride removal from contaminated wastewater by Eskandarpour* et al.* [144] via batch system with respect to changes in initial concentration of fluoride (10–90 mg/L), equilibrium pH (3–11) of sample solution, adsorbent dosage (0.025–0.10 g/50 mL) and co-existing ions (nitrate, chloride, sulfate and phosphate). The Schwertmannite adsorbent had the ability to lower the fluoride concentration to acceptable levels and was regenerable, and therefore can be applied to polish wastewater after a precipitation/coagulation process. Only acidic pH range is considered, as would be expected of fluoride-containing industrial wastewater. Between the pH = 3.0 and 3.7, the uptake of fluoride increases with an increase in pH. Maximum adsorption occurs at pH = 3.7. pH > 3.7, the increase in pH causes the fluoride removal efficiency to decrease sharply.

### 3.2. Biosorbents

Biosorption is an emerging technique for water treatment utilizing abundantly available biomaterials. Various biosorbents have been developed for fluoride removal. Chitin and chitosan are attractive adsorbents because of their unique properties like biodegradability, biocompatibility and low cost, in addition to their particular physical and mechanical properties, resulting from the presence of chemical reactive groups (hydroxyl, acetamido or amino functions) in polymer chains [176].

In the sorption process, chitosan is often used in the form of flakes or powders which are less stable and cause a significant pressure drop which would affect filtration during field applications. The defluoridation capacity of the unmodified chitosan was found to be minimum. These disadvantages outweigh its advantages of biodegradability and indigenousness. If chitosan has been suitably modified into a form which could overcome the above mentioned challenges, then definitely it would throw more light on the field of defluoridation. Chitosan beads which have negligible defluoridation capacity have been chemically modified by introducing multifunctional groups, *viz.*, NH_3_^+^ and COOH groups by means of protonation and carboxylation in order to utilize both amine and hydroxyl groups for fluoride removal. The protonated carboxylated chitosan beads showed a maximum defluoridation capacity of 1664 mg/kg whereas raw chitosan beads displayed much lower adsorption capacity of 52 mg/kg. The sorption process was found to be independent of pH and slightly influenced by the presence of other common anions [177].

The lanthanum incorporated chitosan beads were synthesized for defluoridation use by optimization of various synthesis parameters. These beads showed excellent fluoride removal efficiency of 97% at pH = 5 and overcome the drawbacks associated with the conventional adsorbents [178]. Thakre* et al.* [179] also reported the metal binding characteristic of chitosan to incorporate La(III) to develop a new material and to study various parameters for the synthesis of lanthanum incorporated chitosan beads which alter the properties and fluoride adsorption capacity of lanthanum incorporated chitosan beads. The lanthanum incorporated chitosan beads not only have much higher fluoride adsorption capacity but also has numerous advantages, namely: relatively fast kinetics, high chemical and mechanical stability, high resistance to attrition, negligible lanthanum release, suitability for column applications,* etc.* Lanthanum incorporated chitosan beads can reduce the fluoride concentration in water below the permissible level of 1.5 mg/L and therefore can be used as an effective adsorbent for the defluoridation of drinking water. Kamble* et al.* [113] conducted defluoridation studies using chitin, chitosan and 20% lanthanum incorporated chitosan. Lanthanum-chitosan adsorbents show excellent removal of fluoride from water, which is much better than bare chitosan and chitin. The adsorption of fluoride on the surface of the adsorbent is found to depend mainly on the pH of the solution as well as the concentration and type of co-anions. It was found that the presence of anions has a deleterious effect on the adsorption of fluoride, particularly carbonate and bicarbonate anions. The adsorption of fluoride at acidic pH > 5 was high as compared to alkaline pH. It is a well-known fact that the N in the NH_2_ group of chitosan acts as an electron donor and it is responsible for selective chelation with metal ions (La^3+^, Ce^4+^, Fe^3+^, Ti^4+^, Al^3+^,* etc.*) [180]. Ma* et al.* [181] studied the mechanism sorption of fluoride ions on magnetic-chitosan particle from the water solution in the batch system. The obtained results showed that the maximal amounts of adsorbed fluoride were 20.96–23.98 mg/g. The metal-binding property of chitosan is used to incorporate titanium metal and applied as an adsorbent for fluoride adsorption by Jagtap* et al.* [115]. Titanium macrospheres shows excellent fluoride removal capacity, which is very high compared to chitosan. The adsorption of fluoride on titanium macrospheres mainly depends on the pH and the existing co-ions present in the water. In alkaline pH > 7, fluoride uptake is very low compared to acidic pH. The presence of other co-anions, particularly carbonate and bicarbonate in water has negative effects on fluoride uptake. The biosorbent derived from shell fish processing waste is considered to be one of the key aspects of ecological engineering to suit domestic, biological and environmental conditions.

The applicability of neodymium-modified chitosan as adsorbents for the removal of excess fluoride ions from water was investigated by Yao* et al.* [176]. The effect of various physic-chemical parameters such as temperature (10–50 °C), pH (5–9), adsorbent dose (0.2–2.0 g/L), particle size (0.10–0.50 mm) and the presence of co-anions (NO^3^^−^, Cl^−^ and SO_4_^2^^−^) on removal of fluoride ions were studied. The treatment conditions were optimized: pH value was 7, water temperature was at 323 K, and particle size was 0.10 mm. A salt rejection against the water containing 20 mg/L of fluoride ions was 98.15% at the dosage of adsorbent was only 2.0 g/L, 500 mg/L of chloride, 500 mg/L of sulfate 50 mg/L of nitrogen of nitrate in water respectively, it had no significant effect on the removal rate of fluoride. The equilibrium sorption data were fitted reasonably well for Langmuir isotherm model. The maximum equilibrium sorption was 22.38 mg/g at 30 °C.

Sujana* et al.* [116] synthesized a new biopolymer beads, composite of hydrous ferric oxide (HFO) and alginate, characterized and examined it, with aim of fluoride removal from water. The beads (0.8–0.9 mm) were characterized by chemical analysis, BET surface area, pH_PZC_ and XRD analysis, while the optimum conditions for fluoride removal were determined by studying operational variables *viz.*, pH, contact time, initial fluoride concentration, bead dose, temperature and presence of other anions such as sulfate, phosphate, nitrate, chloride and bicarbonate. Efficiency of fluoride removal by HFO doped calcium alginate beads was tested at different pH values (3.5–9) with 50 mL of fluoride solution (initial fluoride concentration 10 mg/L), bead dose of 1.3 g/L and contact time of 6 h. Over 60% of fluoride was removed from water at pH < 5 and authors concluded that pH had significant effect on fluoride adsorption efficiency of the beads,* i.e.*, fluoride removal was more efficient in pH range from 3.5 to 5 and >5 showed efficiency decreasing trend. BET analysis showed that specific surface area of used biopolymer beads was 25.8 m^2^/g and a pH_PZC_ of 5.15. The study also revealed that SO_4_^2^^−^, PO_4_^3^^−^ and HCO_3_^−^ anions interfere with fluoride adsorption more than NO_3_^−^ and Cl^−^.

Fluoride adsorption capacity and the effects of various parameters such as contact time, pH, adsorbent dose, initial fluoride concentration and co-ions on fluoride adsorption were studied using La-Ce-modified chitosan and La(III)-modified chitosan by Liang* et al.* [98]. The structure characterizations of synthesized adsorbents were conducted using FTIR and XRD. Authors reported the fluoride adsorption capacities, measured during two hours adsorption, of 3.72 mg/g for the La-Ce-modified chitosan and 3.16 mg/g for the La(III)-modified chitosan. They also found out that the presence of co-ions such as bicarbonate and carbonate greatly affected the fluoride adsorption from water.

Davila-Rodriguez* et al.* [118] reported a detailed fluoride adsorption study in packed columns with chitin and chitin-based biocomposite. An empty bed contact time (EBCT) of 20 min was determined as adequate. The initial fluoride concentration of the artificially prepared influent was 5.1 mg/L. About 200 and 300 bed volumes of contaminated water were treated before saturation of packed columns with biocomposite or chitin, respectively. Fluoride was desorbed from the fluoride exhausted chitin and biocomposite by using a NaOH solution as eluent: the regeneration efficiencies were 85% and 84% for chitin and biocomposite, respectively. Authors also examined continuous fluoride adsorption using natural fluoride contaminated water (initial fluoride concentration 3.9 mg/L). The chitin and biocomposite fluoride selectivity was determined as follows: SO_4_^2−^ > HCO_3_^−^ > F^−^ > Cl^−^ > NO_3_^−^. Adsorption capacity at the saturation point (*i.e.*, 200 bed volumes) was 1.7 mg/g for the chitin-based biocomposite, while that for chitin (at 300 bed volumes) it was 3.4 mg/g. Results of this study confirmed the potential of chitin-based biocomposites as adsorbents materials of fluoride and other anions present in water.

Swain* et al.* [119] reported result of water defluoridation using a hybrid material of (Fe/Zr)-alginate microparticles. Various physico-chemical parameters such as equilibrium contact time, pH, initial fluoride concentration and adsorbent dose, were studied in batch adsorption experiments conduced with 50 mL of fluoride solution. Initial fluoride concentrations were ranging from 2 mg/L to 50 mg/L, pH was in a range from 2.0 to 12.0 and adsorption time was ranging from 0 min to 260 min. The desorption characteristic of the hybrid material shows that nearly 89% of fluoride could be leached out at pH = 12, while the maximum removal of fluoride was observed in 210 min at pH = 6.0. The result indicated that the maximum sorption capacity (Fe/Zr)-alginate microparticles was 0.981 mg/g.

Biosorption offers advantages of high efficiency in dilute effluents and no nutrient requirements. Recently considerable interest was observed on the application of biosorbent materials for removal of various pollutants. It provides a cost-effective solution for water management. The biomass of the natural plant *Tinospora cordifolia* demonstrated a good capacity of fluoride biosorption, highlighting its potential for the drinking water treatment process [182]. pH had a strong effect on biosorption capacity and the optimum pH was found to be 7. The biosorption was rapid and equilibrium was achieved within 120 min. The uptake capacity of fluoride was found to be 25 mg/g. The results indicate that Langmuir and Freundlich sorption models were good agreement with the experimental results. Further, the biosorbents were characterized by FTIR spectral analyses. The biomass was found to be very efficient, instantaneous and economical for removing fluoride from drinking water. This biosorptive material is very useful to reduce fluoride within standard WHO permissible limit (1.5 mg/L) at neutral pH.

Fungal biosorbent prepared from *Fusarium moniliforme* for removal of fluoride was investigated by Merugu* et al.* [183]. The extent of defluoridation was dependent on the initial pH of fluoride containing water and decreased with increasing pH. Fluoride removal capacity was found to be 24% at pH = 5.0 and 11% at pH = 8.0. The capacity of fluoride removal decreased with increased bicarbonate concentration, but was independent of the presence of chloride and sulfate. The kinetics of fluoride removal exhibited a rapid phase of binding for a period of 1 h and a slower phase of binding during the subsequent period.

A new type of adsorbent was prepared from orange waste by a simple method of saponification reaction with lime water and used for the removal of some metal ions and, after that, for fluoride removal from water [184]. The rare earth metals ions (Sm(III), Ho(III), La(III), Sc(III) and Lu(III)) were successfully adsorbed on orange waste-adsorbent while adsorption of fluoride ion from water was investigated using this adsorbent after being loaded with above-mentioned rare earth metal ions. It was found that fluoride adsorption was fast and dependent on solution pH. The maximum adsorption capacity for fluoride was evaluated as 0.60, 0.92, 1.06 and 1.22 mmol/g for Sc(III), Ho(III), La(III) and Sm(III) loaded adsorbent, respectively. The La(III) and Sm(III) loaded orange waste-adsorbent showed stronger interaction with fluoride ion even at trace concentrations, suggesting that these materials can be employed as an effective adsorbents.

Sivasankar* et al.* [138] synthesized a hybrid cerium-impregnated adsorbent using potato starch as a source of carbon. Synthesized adsorbent was used for defluoridation of synthetic water and the effects of fluoride initial concentration (2.8–8.3 mg/L), temperature (25–45 °C), adsorbent dose (25–150 mg/L), pH (5.5–9.0) and coexisting anions (Cl^−^, HCO_3_^−^, NO_3_^−^, SO_4_^2−^ and PO_4_^4−^) were studied. The highest adsorption capacity of 29.1 mg/g was achieved at pH = 7.75 and lowest tested initial fluoride concentration of 2.8 mg/L, while further increase of initial fluoride concentration decreased the uptake capacity of used strach-based adsorbent.

### 3.3. Geomaterials

In recent years, much effort has been devoted to the investigation and development of other more cost-effective fluoride sorbents. Wang* et al.* [185] reported that geomaterials can be cost-effective fluoride sorbents for use in water treatment. They investigated a heavily-weathered Tertiary soil from Xinzhou, Shanxi, China as a sorbent for defuoridation of high fluoride drinking water. The soil is composed of quartz, feldspar, illite and goethite, with a Fe-oxide content of 6.75%. Various heating temperatures were applied, ranging from 100 °C to 900 °C in 100 °C increments and concentrations of fluoride were between 0 mg/L and 60 mg/L. Gopal* et al.* [186] and Yadav* et al.* [187] studied the removal of fluoride from water using sawdust, groundnut husk and sand. The obtained results indicate that these chemically treated natural adsorbents remove fluoride effectively. Fan* et al.* [78] investigated the adsorption kinetics and adsorption capacity of low cost materials at a low initial fluoride concentration. The experiments were carried out at a neutral pH, and radioisotope ^18^F rather than ^19^F was used since ^18^F can be rapidly measured by measuring the radioactivity with a resolution of 1 × 10^−13^ mg or 0.01 μCi. The tested materials were fluorspar, calcite, quartz and quartz activated by ferric ions. Fluorspar can remove about 25% of fluoride from solution with an initial fluoride concentration range from 2.5 × 10^−5^ mg/L to 6.34 × 10^−2^ mg/L. Calcite can remove 12% of fluoride and is only better than quartz. The activation of ferric ions on quartz can significantly improve the adsorption capacity of quartz. After the activation, the fluoride removal increased from 5.6% to 20%. Ferric ion was adsorbed on quartz surface and acted as a bridge between quartz and fluoride.

Clays have attracted considerable interest as potential candidates of carrier materials due to their inexpensiveness, availability, environmental stability and high surface area/sorption capacity, anti-ultraviolet-ray effects and ion exchange properties. Attapulgite is a crystalline hydrated magnesium aluminum silicate with unique three dimensional structures and has a fibrous morphology, it has permanent negative charges on its surface, which enable it to be modified by cationic surfactants, to enhance contaminant retention and retard contaminant migration [188]. Guo and Tian [69] reported that anion clay, hydrocalumite, is capable of fluoride and arsenic removal from water via both precipitation and sorption at a wide range of initial solution fluoride and arsenate concentrations. The effect of contact time on fluoride and arsenate removal was investigated with 0.3, 10 and 20 mmol/L of fluoride solutions and impact of common co-existing anions (HCO_3_^−^, SO_4_^2−^ and Cl^−^) on solution defluoridation was evaluated by using 1:10 milliequivalent ratio of fluoride to these competitive ions. The authors concluded that hydrocalumite is a good adsorbent for treatment of high fluoride water and initial fluoride concentrations could be reduced to below the corresponding drinking water standards of 1.5 mg/L. The authors emphasized that fluoride removal from water by hydrocalumite involved mechanism of dissolution-reprecipitation and anion exchange, and the contribution of fluorite precipitation were induced by the release of Ca^2+^ due to hydrocalumite dissolution rises with increase of initial solution fluoride concentration. Solution pH value has little effect on defluoridation by hydrocalumite due to its pH buffering effect, while the coexistence of competitive anions and the variation of reaction temperature affected the fluoride removal significantly. The maximum fluoride uptake capacity of hydrocalumite was 719.1 mg/g.

Guo and Reardon [150] investigated fluoride removal from water using mechanochemically synthesized anion clay—meixnerite, and calcined meixnerite regarding initial fluoride concentration, contact time and effect of sulfate and bicarbonate anions. Batch sorption experiments were conduced with initial fluoride concentrations ranging from 12.4 mg/L to 248.0 mg/L, molar ratio of fluoride in solution to solid varying from 0.1 to 2.0, and contact time ranging from 0.5 h to 50 h. The experimental results show that efficient fluoride removal was obtained using calcined meixnerite and over 95% fluoride was removed for all concentrations up to 75 mg/L. Mechanism of fluoride removal via uncalcined and calcined meixnerite was investigated by XRD and SEM. The SEM images of reacted meixnerite samples show only needle-like crystals in the uncalcined material, whereas both grain-like and layered crystals are present in the calcined material. The XRD patterns for reacted samples of calcined meixnerite indicate the precipitation of secondary fluoride-containing phases at initial fluoride concentrations ≥ 74.4 mg/L, which is the basic mechanism for the improved ability of calcined meixnerite to remove fluoride compared to uncalcined meixnerite. The higher fluoride uptake by calcined meixnerite is due to greater availability of fluoride to its interlayer sites, since interlayers were generated during reaction of the F-containing solution with the calcined material and some F^−^ did not have to diffuse from the solution into the interlayers to replace existing OH^−^ ions as it did for the uncalcined meixnerite. The authors also emphasized that fluoride diffused from solution to intraparticle active sites and that chemical sorption on active sites of in uncalcined meixnerite was much slower than in calcined meixnerite.

Zirconium-attapulgite adsorbent has been synthesized and used for fluoride adsorption. The fluoride adsorption capacity of the Zr-attapulgite adsorbent was higher in comparison with attapulgite, which was due to the changes of the surface charge of the adsorbent and the generation of abundant hydroxyl ions. The Zr-attapulgite adsorbent exhibited good performance for fluoride removal over a wide pH range of 3.70–7.50. Adsorption of fluoride reached the equilibrium in 110 min for different initial fluoride concentration. The equilibrium data were better represented by the Langmuir isotherm than the Freundlich isotherm. The adsorption process followed the pseudo-second-order model for fluoride, fluoride adsorption was influenced by the phosphate, sulfate and bicarbonate ions, but not by chloride and nitrate ions. The results of desorption and reuse experiments indicated that the Zr-attapulgite adsorbent could be employed as a promising adsorbent for fluoride adsorption from drinking water [189].

The ability of three Tunisian clays to remove fluoride from acidic waste solution has been investigated. The studies were carried out as functions of solid-liquid percentage (10%, 20% and 30%), contact time (0–190 min), pH and effect of concentrations of other ions. The kinetic study shows that the percentage of fluoride removed increased with the agitation time [190].

Bentonite is sediment (usually) composed of smectite. The bentonites are widespread in most continents of the world and its low cost makes it a strong candidate as an adsorbent for the removal of many pollutants from wastewaters. Bentonite can sorb fluoride in the acid conditions and has pH dependency. Removal of fluoride from aqueous solution using granular acid-treated bentonite was studied by batch and column adsorption experiments. The results of the batch adsorption experiments demonstrated that the maximum fluoride removal was obtained at pH = 4.95 and it took 40 min to attain equilibrium. Kinetics data fitted the pseudo-second-order model. The adsorption of fluoride by granular acid-treated bentonite in batch systems can be described by the Freundlich isotherm, and the adsorption capacity was 0.094 mg/g [191].

Modification of bentonite clay with an electro-positive atom (lanthanum, magnesium and manganese ions), in order to enhance its adsorption capacity for fluoride ions from drinking water, was conducted by Kamble* et al.* [192]. La-bentonite shows the 10% higher fluoride adsorption capacity as compared to bare bentonite as well as the other chemically modified clays. The adsorption of fluoride on the surface of the adsorbent is found to depend mainly on the pH of the solution as well as the concentration and type of co-ions. The adsorption of fluoride at pH > 5 was high as compared to alkaline pH, which also indicates its fluoride uptake mechanism through exchange of hydroxyl ions from adsorbent surface. The reasonable adsorption capacity and positive effect of co-ions suggest the potential of such materials in the defluoridation of water.

The removal of fluoride from aqueous solution by using montmorillonite was studied by a batch equilibration technique. Influences of contact time (10–240 min), pH (2–10), initial fluoride concentration (2–100 mg/L) and adsorbent dosage (1–10 g/L) on the adsorption were investigated. It was found that the sufficient time for adsorption equilibrium of fluoride ions is 180 min. The maximum removal of fluoride ion was obtained at pH = 6. The mechanism for fluoride removal was explained by considering the interaction between the metal oxides at the surface of montmorillonite and fluoride ions. The adsorption isotherms were analyzed using the Langmuir, the Freundlich, and the three-parameter Redlich-Peterson isotherms. The adsorbed fluoride could be easily desorbed by washing the adsorbent with a solution pH = 12 [193].

Pumice is a light, porous, volcanic stone with a large surface area. It is easily and cheaply found in nature or some kinds of waste. Pumice is composed of highly microvesicular glass pyroclastic with very thin, translucent bubble walls of extrusive igneous rock. Pumice has an average porosity of 90%, and initially floats on water. Malakootian* et al.* [101] recommended pumice as effective and low cost absorbent for fluoride removal from aqueous solutions. They examined efficiency of fluoride removal from synthetic fluoride solution and Kuhbonan water which contain elevated fluoride concentration. The effect of pH, contact time, initial fluoride concentration and adsorbent dose on the fluoride sequestration was investigated with fluoride solution and estimated optimum conditions were studied as a case study. The results showed that increasing of the absorbent amount; contact time and pH improve the efficiency fluoride removal. The maximum fluoride uptake was obtained in pH = 7.0 during 180 min of adsorption process. The authors also concluded that increase of initial fluoride concentration decrease efficiency of fluoride removal. Due to the Freundlich isotherm and pseudo second order kinetic, the maximum adsorption capacity and constant rate were found to be 0.31 mg/g and 0.21 mg/g, respectively. This study also showed that, at optimum conditions, 74.64% of the initial fluoride concentration can be reduced from natural fluoride contaminated water.

It has been widely tested and used in water treatment as an adsorbent, filter bed and support media. Asgari* et al.* [108] used pumice that is functionalized by the cationic surfactant, hexadecyltrimethyl ammonium as an adsorbent for the removal of fluoride from drinking water. This work was carried out in two parts. The effects of hexadecyltrimethyl ammonium loading, pH (3–10), reaction time (5–60 min) and the adsorbent dosage (0.15–2.5 g/L) were investigated on the removal of fluoride as a target contaminate from water through the design of different experimental sets in the first part. The results from this first part revealed that surfactant-modified pumice exhibited the best performance at dose 0.5 g/L, pH = 6, and it adsorbs over 96% of fluoride from a solution containing 10 mg/L fluoride after 30 min of mixing time. The four linear forms of the Langmuir, Freundlich, Temkin and Dubinin-Radushkevich isotherms model were applied to determine the best fit of equilibrium expressions. The maximum amount of adsorption was 41 mg/g. The kinetic studies indicated that the adsorption of fluoride best fitted with the pseudo-second-order kinetic. Accordingly, the surfactant-modified pumice was shown to be an efficient adsorbent for the removal of fluoride and a promising option for water fluoride treatment. However, further studies will be required to scale up and optimize process variables. Salifu* et al.* [109] also investigated the possibility of fluoride removal from model water using aluminum oxide coated pumice. Initial fluoride concentrations were between 1.5 mg/L and 5.0 mg/L and adsorption time was approximately 1 h, while adsorbent dose was 10 mg/L. The equilibrium adsorption of fluoride by aluminum oxide coated pumice conformed reasonably to five isotherm models in the order: Generalized model > Langmuir type 2 > BET > Temkin > Dubinin-Radushkevich; with a maximum capacity of 7.87 mg/g. Efficient fluoride removal was obtained within the pH range of 6–9, which makes it possible to avoid pH adjustment of treated water which implies additional cost and operational difficulties during water treatment. Based on the results of kinetic adsorption experiments, authors emphasized that at a neutral pH = 7.0 which is a more suitable condition for groundwater treatment, fluoride adsorption by aluminum oxide coated pumice was faster in the initial period of contact.

In order to enhance fluoride adsorption capacity, Sepehr* et al.* [194] modified surface of pumice using aqueous solution of magnesium chloride and hydrogen peroxide. Untreated pumice and two other it modifications were tested in range of pH from 2 to 10, using adsorbent in a doses from 2 g/L to 10 g/L in fluoride solutions with initial concentration ranging from 5 mg/L to 20 mg/L at different temperature (10–50 °C). The competency effect of co-ions (nitrate, chloride, magnesium, calcium and sulfate) was also studied. The authors reported that the maximum fluoride uptake capacities of 65.4% for untreated pumice, 68.4% for pumice modified with magnesium chloride and 70.8% for pumice modified with hydrogen peroxide were observed at pH = 6. The highest value of adsorption capacity due to Langmuir isotherm model of 11.765 mg/g was calculated for pumice modified with hydrogen peroxide.

Zeolites are a class of natural materials that can be effectively used as adsorbents due to their cation-exchange characteristics, although Samatya* et al.* [195], investigating the adsorption characteristics of zeolites, emphasized that the limited use of zeolites as fluoride adsorbents is probably due to the fact that zeolites usually have negative surface charges at all pH values, which causing a high adsorption capacities for cations, but low for anions because of electrostatic repulsions when anions approach the negatively-charged zeolite surface. Therefore, many researchers modified zeolite surface with multi-valent metallic cations with the aim of increasing the adsorption capacity of zeolites [155]. Three main methods are proposed for modifications of zeolites including skeleton element modification, non-skeleton element modification and surface modification [196].

Gómez-Hortigüela* et al.* [157] tested the fluoride removal from drinking water using eleven samples of naturally-occurring zeolites in Ethiopia (Tigrae region). Adsorption characteristics of raw, untreated zeolites, were preliminary tested, and zeolite with higher adsorption capacity was examined further. Using chosen zeolite, constituted of analcime and mordenite, at a dose of 200 g/L in fluoride solution with initial fluoride concentration of 18.3 mg/L, authors obtained residual fluoride concentration of 1.9 mg/L (90% fluoride removal). Using different initial fluoride concentrations ranging from 20 mg/L to 200 mg/L, zeolite showed adsorption capacity ranging from 0.21 mg/g to 0.08 mg/g, while after several consecutive uses, the maximum defluoridation capacity was 0.47 mg/g. Desorption test, conduced with NaOH, showed efficient regeneration of exhausted adsorbent (87% of fluoride ions can be desorbed), although such treatment leads to a partial regeneration of the adsorbent (recovers up to 56% of the initial defluoridation capacity). Using real water sample with high fluoride concentration (9.7 mg/L, pH = 8.5) and adsorbent dose of 100 g/L during contact time of 20 h, 76% of fluoride was removed and a final fluoride concentration was 2.4 mg/L (defluoridation capacity of 0.07 mg/g).

Although some zeolites have satisfactory adsorption capacities, researchers preferred to modify their surface. Sun* et al.* [156] modified natural stilbite zeolite, from the northern part of China, with Fe^3+^, and tested its adsorption capacities. Batch adsorption experiments were conduced to investigate the effect of contact time, adsorbent dose, initial fluoride concentration, pH and coexisting ions. Adsorbent characterization was also conduced using energy-dispersive X-ray (EDX) and X-ray photoelectron spectroscopy (XPS). Authors reported that fluoride uptake increases with contact time and reaches equilibrium within 2 h when maximum efficiency of fluoride removal is about 92%. Increase of adsorbent doses showed a positive effect on fluoride removal due to the higher number of active adsorption sites, and dose of 10 g/L was determined as minimum dosage for maximum fluoride removal and the dose at which it is possible to achieve the residual fluoride concentration of 1 mg/L (initial concentration was 10 mg/L) The maximum adsorption capacity of ferric-stilbite zeolite due to Langmuir adsorption model was reported to be 2.31 mg/g at pH = 6.94. XPS and EDX studies all reveal that Fe^3+^ is impregnated on the natural stilbite zeolite and the fluoride is adsorbed on the ferric-stilbite zeolite. The desorption and regeneration tests showed that used adsorbent can be regenerated with HCl.

Teutli-Sequeira* et al.* [197] determined and compared adsorption characteristics of three modified adsorbent—hematite, zeolite from Oaxaca, Mexico and calcite from Zacatecas, Mexico. Listed adsorbents were electrochemically modified with aluminum and experiments were conduced using fluoride solutions with different initial concentrations and drinking water containing naturally 8.29 mg of fluoride ions per liter during different contact times ranging from 50 min to 72 h and adsorbent doses ranging from 0.02 g to 0.2 g. The highest adsorption capacity was found for aluminum-modified zeolites at adsorbent dosage of 10 g/L (10.25 mg/g for aqueous solutions with initial fluoride concentration of 9 mg/L and 1.16 mg/g for drinking water with naturally elevated fluoride concentration), when the residual fluoride concentrations were reported to be 0.08 mg/L and 0.7 mg/L, respectively.

Zhang* et al.* [198] investigated defluorination of wastewater by calcium chloride modified natural zeolite from Jinyun, Zhejiang, China. Effects of different operational conditions including the adsorbent dosage (0.5–10 g/100 mL), initial pH value (4–9), temperature (25–45 °C), and contact time (10 min to 12 h) were investigated. Authors reported that the chemical modification of natural zeolite with CaCl_2_ results in enhancement in its fluoride removal efficiency and removal efficiency was increased from maximal 9.6% for untreated zeolite to 94.3% for calcium chloride modified zeolite (adsorbent dose of 100 g/L, initial fluoride concentration 70 mg/L, contact time 6 h, initial pH = 6.0, temperature 25 °C). Maximum fluoride adsorption capacity of modified zeolite was found to be 1.766 mg/g. Experiments of co-existing ions effect showed that chloride and sulfate ions did not affect the fluoride removal efficiency, while a significant decrease fluoride adsorption was noted in the presence of carbonate ions due to its competition for active sites on an adsorbent surface.

Rahmani* et al.* [154] investigated and compared adsorption capacities of unmodified and modified zeolite from Miyaneh region, Iran. Used zeolite (clinoptilolite) was modified with trivalent metal ions (Al^3+^ and Fe^3+^). The effects of different contact time (2–24 h), initial fluoride concentration (0.5–4 mg/L), pH value (4–10) and co-existing ions (bicarbonate, chloride and sulfate) on fluoride removal were investigated. Authors reported that untreated zeolite showed very low adsorption efficiency (less than 20% of fluoride was removed) while aluminum- and ferric-modified zeolite showed nearly 76% and 65% of fluoride uptake from solution with initial fluoride concentration of 5 mg/L, while the equilibrium conditions was reached after 20 h. Increasing the initial fluoride concentration negatively affected the percentage of fluoride removal. Results also showed that pH and bicarbonate content strongly influenced fluoride adsorption and effectively fluoride removal was obtained at acidic pH since bicarbonate ions causing a higher pH and diminished the affinity of the adsorption sites for fluoride. Comparing the adsorbents efficiency in fluoride removal from naturally fluoride-contaminated and model solution, authors observed that the existence of ions in natural water reduced the absorption rate of fluoride. Aluminum-modified zeolite showed the best adsorption characteristics.

Onyango* et al.* [76] investigated the fluoride sorption characteristics of zeolite F-9 containing surface-active sites created by exchanging Na^+^-bound zeolite with Al^3^^+^ or La^3^^+^ ions. The authors investigated effect of pH and bicarbonate content on fluoride removal and reported that sorption on aluminum-modified zeolite was consistent with an ion-exchange mechanism, while sorption on lanthanum-modified zeolite was of a physical nature involving electrostatic interaction. They also noted that the higher pH diminished the affinity of the active sites for fluoride. After testing the efficiency of modified zeolite using naturally fluoride-contaminated groundwater authors concluded that aluminum-modified zeolite was found to be superior to lanthanum-modified zeolite in fluoride uptake.

Modification of bentonite clay with an electro positive atom (using lanthanum, magnesium and manganese), in order to enhance its adsorption capacity for fluoride ions from drinking water, was conducted by Kamble* et al.* [192]. Ten percent La-bentonite shows higher fluoride adsorption capacity as compared to bare bentonite as well as the other chemically modified clays. The adsorption of fluoride on the surface of the adsorbent is found to depend mainly on the pH of the solution as well as the concentration and type of co-ions. The adsorption of fluoride at pH > 5 was high compared to alkaline pH, which also indicates its fluoride uptake mechanism through exchange of hydroxyl ions from the adsorbent surface. The reasonable adsorption capacity and positive effect of co-ions suggest the potential of such materials in the defluoridation of water.

The modification effects of hematite with aluminum hydroxide were investigated by Teutli-Sequeira* et al.* [103] unmodified hematite is not an efficient material for fluoride adsorption from aqueous solutions; however, hematite modified with aluminum hydroxide is an efficient adsorbent for the removal of fluoride ions from water. The optimum pH range for maximum adsorption was between 2.34 and 6.26 and the sorption capacities of modified hematite for fluoride ions increased by increasing the sorbent dosage from 0.06 g to 0.18 g.

Shan and Guo [104] examined fluoride adsorption on modified natural siderite. Modified natural siderite has been obtained using different calcification temperature (300–450 °C), calcination time (0.5–5 h) and the mixing ratio of natural siderite powder, Al_2_(SO_4_)_3_ and AlOOH. Adsorption characteristics for fluoride removal on the modified siderite were evaluated with batch and column experiments. Batch experiments were conducted using solutions with different initial fluoride concentrations (2–25 mg/L), pH (2–12) at different temperature (15–45 °C). The effect of coexisting anions (Cl^−^, SO_4_^2−^, NO_3_^−^, HCO_3_^−^ and PO_4_^3−^) was also examined. The column study was conducted using solution with initial fluoride concentration of 3 mg/L. Authors reported that the maximum adsorption capacity of 4.42 mg/g was obtained with modified natural siderite made by mixing of natural siderite powder, Al_2_(SO_4_)_3_ and AlOOH with the mass ratio of 50:0.3:10 with was calcinated at 450 °C for 3 h. A significant effect of pH on fluoride removal was not detected, while the coexistence of bicarbonate and phosphate ions had a negative effect on fluoride adsorption onto modified siderite. Examining the performance of regenerated modified siderite adsorbent, authors noted its lower adsorption capacity compared to the fresh one.

Bauxite is one of the abundantly available mineral, mainly consists of oxides of alumina, iron, silica and titanium. Conducting the experiments on water defluoridation with bauxite, Sajidu* et al.* [199] reported that up to 93.8% fluoride was removed from fluoride solution with initial concentration of 8 mg/L when adsorbent dose of 2.5 g/200 mL were used. Powder XRD characterization of the raw bauxite showed gibbsite (Al(OH)_3_) and kaolinite (Al_2_Si_2_O_5_(OH)_4_) as the major components. The high defluoridation capacity of the bauxite is thus attributable to gibbsite and kaolinite minerals.

Sujana* et al.* [200] investigated the adsorption efficiency of bauxite for fluoride removal from synthetic as well as ground water samples. The adsorption of fluoride was highly dependent on pH, temperature and initial adsorbate and other anion concentrations in the solutions. The optimum pH range for fluoride on bauxite surface was found to be 5–7, which makes it suitable for water treatment. The kinetic study reveals that the fluoride adsorption on bauxite surface followed first order kinetics. The Langmuir adsorption capacity was found to be 5.16 mg/g.

### 3.4. Carbonaceous Materials

In recent years, various carbons from different species have been prepared and were used as adsorbents for defluoridation studies as well as for other purposes. The raw materials used for the preparation of carbon are readily available. Any carbonaceous materials (animal, plant, or mineral origin) with high concentration of carbon can be simply changed into activated carbon (using both chemical or gas activation methods). The most common raw materials are wood, charcoal, nut shells, fruit pits, brown and bituminous coals, lignite, peat, bone and paper mill waste (lignin), synthetic polymers like PVC, are used for the manufacture of activated carbon. Activated carbon obtained from hard wood is preferable for adsorption because charcoal obtained from soft wood, such as pinewood, is very unstable and readily crumbles. Activated carbons are commonly prepared by two basic processes: physical or gas activation and chemical activation. The choice of activation method also depends on the starting material and whether a low or high density, powdered or granular carbon is desired [201]. Activated carbons produced by carbonization employing slow substrate heating in the absence of air below 600 °C. This removes volatiles, and chemical or physical activation follows. Treatment with oxidizing agents (steam, carbon dioxide, or oxygen) at elevated temperature or with chemical activants (ZnCl_2_, H_2_PO_4_, H_2_SO_4_, KOH, K_2_S, KCNS,* etc.*) completes the activation.

Chemical activants may promote crosslinking, forming a rigid, less volatile matrix with smaller volume contraction at high temperature. An advantage of chemical activation is the lower temperature required. Chemical activation gives higher global yields since char burn-off is not required. Post activation removes residual catalyst, which may be recovered and reused [201,202,203,204].

Although activated carbons are the most used adsorbents worldwide with high capacities, they are generally characterized by low selectivity for fluoride ions due to physical adsorption, so in most studies, prior examination of fluoride removal efficiency, activated carbons are modified by oxidation and subsequent impregnation with high valent ions such as zirconium, titanium, iron, calcium,* etc.*

Oxidation is one of the most conventional modifications used for activated carbons. It is mainly used to introduce carbon–oxygen surface groups in activated carbon. Oxidation methods involve the utilization of oxidizing gases (*i.e.*, oxygen, steam, carbon dioxide,* etc.*) or oxidizing solutions (*i.e.*, nitric acid, hydrogen peroxide, chlorine water,* etc.*) [205]. Treatment of activated carbon with hydrogen peroxide increases the enrichment efficiency relative to non-oxidized activated carbon [206]. Oxidation with nitric acid produces a slight decrease of the porosity and the surface area of activated carbon, although drastic changes in the chemical nature of the surface take place. Heat treatment at 700 °C of the acid-treated carbon produces elimination of the oxygen surface complexes, with only a fraction of CO groups with high thermal stability remaining. However, the surface area and pore volume increase, due to carbon gasification. After oxidation, activated carbon prepared with steam fixed a larger amount of oxygen groups than carbon activated with carbon dioxide as a consequence of the wider microporosity created by steam activation. However, carbons activated with CO_2_ exhibit high surface area and more stability of oxygen surface groups after heat treatment. Finally, date pits could be used as precursors to produce activated carbons with a well-developed porosity and tailored oxygen surface groups for different applications [207].

Activated carbon prepared by one-step steam pyrolysis of rice straw at 550, 650 and 750 °C were modified by liquid-phase oxidation using HNO_3_, H_2_O_2_ and KMnO_4_ was the object of investigation by Daifullah* et al.* [85]. Characterization of these carbons was made by their surface area, porosity, acidity, alkalinity, pH_pzc_, pH and ability to remove fluoride anion. Batch adsorption studies effect pH (2–10), adsorbate concentration (5–20 mg/L), adsorbent dosage (25–500 mg/L), contact time (1–24 h), temperature (25–55 °C), and co-ions (SO_4_^2^^−^, Cl^−^, Br^−^). The authors reported that the best results and 100% of fluoride removal was obtained with activated carbons oxidized with KMnO_4_. They emphasized that the solution pH was the most important parameter affecting adsorption because the optimum pH for maximum adsorption was determined to be 2.0 for fluoride. Sorption occurs better at lower temperatures and the presence of natural organic matter decreases fluoride adsorption. Activated carbon obtained by burning and carbonization of the *Morringa indica* bark possessing appreciable defluoridation efficiency was reported by Karthikeyan* et al.* [208]. The authors reported that over 71% of fluoride was removed from neutral aqueous fluoride solution (2 mg/L). Further increase of fluoride removal decrease adsorption capacity of tested activated carbon.

Batch adsorption dynamics and equilibrium studies for the removal of fluoride ions from aqueous solution using indigenously *Acacia farnesiana* carbon has been carried out under various experimental conditions at room temperature. Results found that, initially, the percentage removal of fluoride ions increased with a decrease in initial concentration and increased with an increase in contact time and, after 40–45 min, the percentage removal is found to be almost constant. Adsorption is highly pH sensitive and the optimum pH range for appreciable or maximum adsorption of fluoride ion is found to be 6.5–7.0, with maximum absorption around 6.9. Results also indicate that the fluoride adsorption reaches a maximum in the pH range of 6.5–7.0, and then decreases with increasing pH. For the same pH, fluoride adsorption follows the Freundlich isotherm, indicating that the *Acacia farnesiana* carbon surface is highly heterogeneous [209]. It is a widely used adsorbent in the treatment of wastewaters due to its exceptionally high surface areas which range from 500 m^2^/g to 1500 m^2^/g, well-developed internal microporosity structure as well as the presence of a wide spectrum of surface functional groups [210].

The removal of fluoride on commercially activated carbon and indigenously prepared activated carbons from *Pithacelobium dulce*, *Ipomoea batatas* and *Peltophorum ferrugineum* have been studied by Emmanuel* et al.* [211]. The results of the experiments have shown that the percentage of fluoride removed increased with the increase of contact time (10–120 min) and dose of adsorbent (0.5–9 g/L). Conversely, the percentage of removal decreased with the increase in initial concentration of the standard fluoride solution (1–8 mg/L). The results suggest that intraparticle adsorption is very important in the adsorption process. The adsorption process is found to be of first order with the intra particle diffusion as one of the rate determining steps. Among the adsorbents under consideration, *Pithacelobium dulce* carbon possesses the highest or the maximum adsorption capacity. Hence it is the best and the most effective adsorbent in the removal of the fluoride content in water. The next in the order on the basis of its efficacy in removing the fatal content is *Ipomoea batatas* carbon and *Peltophorum ferrugineum* carbon. The adsorption capacity and efficacy in the removal of fluoride are far greater than commercial activated carbon.

Alagumuthu* et al.* [83] have investigated the potential of zirconium impregnated cashew nut shell carbon and compared its performance with cashew nut shell carbon for fluoride removal from aqueous solutions time of 180 min was fixed as minimum contact time for the maximum defluoridation of the sorbent. The zirconium impregnated cashew nut shell carbon recorded a maximum percentage of fluoride removal 80.33% when compared with cashew nut shell carbon 72.67%. Fluoride removal by zirconium impregnated groundnut shell carbon has also been evaluated by Alagumuthu and Rajan [82]. The sorption of fluoride ion on groundnut shell carbon and zirconium impregnated groundnut shell carbon has been investigated as a function of contact time in the range of 60–210 min with 3 mg/L as initial fluoride concentration and 2.0 mg of adsorbent at room temperature. The zirconium impregnated carbonized ground nut shell recorded a maximum percentage of fluoride removal, 83.77%, when compared with carbonized ground nut shell which showed fluoride removal of 63.67%. No significant influence on fluoride removal of the material was observed in the presence of Cl^−^ and SO_4_^2−^. However, in the presence of excess HCO_3_^−^, the defluoridation efficiency decreases from 83.77% to 74.6% in presence of about 500 mg/L of bicarbonate. It is observed that with increasing of pH of solution, the fluoride removal by these sorbents decreased. At pH = 3, the maximum percentage of fluoride removal by groundnut shell carbon and zirconium impregnated groundnut shell carbon was 84.67% and 94.33%, respectively.

Alagumuthu* et al.* [73] also examined adsorbent obtained by burning, carbonization and thermal activation of *Cynodondactylon*, and reported appreciable defluoridation efficiency. Fluorides have been removed by 83.77% while keeping 3.0 mg/L fluoride concentration and 1.25 g dosage of adsorbent at neutral pH. The used adsorbents could be regenerated by 67.4% using of 2% sodium hydroxide.

Graphene is a single flat atomic sheet of carbon with the atoms arranged in a two-dimensional (2D) honeycomb configuration [212]. It has drawn much scientific attention since its discovery due to its unique electronic and mechanical properties, specific magnetism, excellent mobility of charge carriers, and high thermal conductivity. It exhibits great promise for potential applications in many technological aspects such as field-effect transistors, solar cells, sensors, and adsorbent for heavy metal removal. The adsorption capacities and rates of fluoride onto graphene at different initial pH, contact time, and temperature were evaluated by Li* et al.* [79]. The experimental results showed that graphene is an excellent fluoride adsorbent with an adsorption capacity of up to 17.65 mg/g at initial fluoride concentration of 25 mg/L and temperature of 25 °C.

The adsorption studies on the removal of fluoride from potable water using activated *Dolichos lab lab* carbon was carried out under various experimental conditions. The results of experiments have shown that the percentage of fluoride removal has increased with the increase of contact time and dose of adsorbent. Among the four activated adsorbent samples prepared from activated *Dolichos lab lab* carbon under consideration, HNO_3_ activated carbon possesses the highest or maximum adsorption capacity. Hence it is the best and the most effective adsorbent sample in the removal of fluoride content in water [213].

Hernández-Montoya* et al.* [86] investigated the possibility to obtain low cost carbon selective for fluoride removal, and, therefore, they impregnated pecan nut shells with a calcium-rich solution extracted from egg shell using L_4_ orthogonal array of the Taguchi method for optimization of the carbon synthesis. After synthesis, obtained low cost carbon was tested for fluoride removal from synthetic and real groundwater samples. The fluoride adsorption capacities of carbon were measured at 30 °C using an adsorbent/solution ratio of 8 g/L and a model solution with initial fluoride concentration of 20 mg/L, while used real groundwater had initial fluoride concentration of 14.10 mg/L. They observed that used synthesized low cost carbon had better adsorption performances when model solution was treated (84% of initial fluoride concentration was reduced), while only 20% of initial fluoride concentration was reduced from groundwater samples when the same low cost carbon was used.

### 3.5. Industrial Products and By-Products

Bone char is a granular material produced by the carbonization of animal bones, consisted of around 10% carbon and 90% calcium phosphate, which has shown good potential defluoridation characteristics. Zhu* et al.* [214] prepared modified bone char by loading Al^3+^ onto/into bone char and its performance for fluoride removal from drinking water was investigated by batch adsorption experiments. The factors of influencing the fluoride removal rate, including the types of the aluminum salt (AlCl_3_, Al(NO_3_)_3_, NaAlO_2_, Al_2_(SO_4_)_3_), the initial fluoride ion concentration (2–20 mg/L), the mass of the adsorbent (0–14 g/L) and the adsorption time (0–72 h), was studied in the research. The results showed that the synthesized adsorbent was effective for the removal of fluoride with relatively fast kinetics. The fluoride removal percentage was 97% by 10 g/L modified bone char in the 10 mg/L fluoride solution at pH = 7.25. There are good prospects for modified bone char in practical applications for the removal of fluoride from contaminated drinking water.

Kang* et al.* [215] investigated cement paste for fluoride removal as abundant and cheaper alternative agent. Cement pastes were prepared by crushing 28 day old cement paste blocks made of an ordinary Portland cement at water to cement ratio of 0.5. The crushed particles were then passed through ASTM standard sieves. Cement paste particles with sizes less than 0.15 mm were used as cement pastes for batch experiments. The cement paste was competitive to lime, a common fluoride removal agent. Various Ca-bearing hydrates such as portlandite, calcium silicate hydrate, and ettringite in the cement paste were identified to remove fluoride by precipitating CaF_2_ and/or adsorbing F^−^ ions. In the batch slurry experiments using cement paste and lime simultaneously, 50%–67% of lime can be substituted by cement paste to satisfy fluoride effluent limitation of 15 mg/L. Fluoride removal reactions in cement paste slurries were strongly affected by pH, and an optimal pH for the cement paste slurries exists between 7.0 and 11.5. From the result of the column experiment to observe the fluoride removal capacity of cement paste, the hydrofluoric acid wastewater concentration of 1150 mg/L was immediately reduced to less than 15 mg/L. These results indicate that cement paste generally has advantageous characteristics as an economical and viable substitute for lime to remove fluoride. From the evaluation of Ca-bearing materials (cement, cement paste, lime), cement paste was found to have a substantial removal capacity for fluoride thus it has the potential to be developed into an abundant and cheaper alternative agent.

Waste mud is one of the most promising adsorbents due to its zero price and easy availability. Waste mud emerges as an undesired by-product during industrial applications. These wastes represent unused resources and in some cases present serious disposal problems so the utilization of waste mud for removal of impurities is an important application. Three different forms of waste mud were tested for their fluoride removal performance: original waste mud, acid-treated waste mud, and precipitated waste mud [216]. Precipitated waste mud exhibited greater performance than the others. Adsorption studies were conducted as a function of pH (2–8), contact time (0–480 min), initial fluoride concentration (0–900 mg/L), adsorbent concentration (1–15 g/L), temperature (0–40 °C),* etc.* Studies were also performed to understand the effect of some co-existing ions present in aqueous solutions. The adsorption process was independent of pH for all types of waste mud. Maximum fluoride uptake was obtained with precipitated waste mud (27.2 mg/g). The kinetic study indicated that adsorption of fluoride was very rapid, and the equilibrium was reached within 60 min at room temperature.

Adsorption studies using red mud from alumina refineries as unconventional adsorbent for water and wastewater treatment purposes are motivated by the fact that red mud is a fine-grained mixture of oxides and hydroxides, capable of removing several contaminants, as well as being widely available. Removal of fluoride from water using granular red mud according to batch and column adsorption techniques is described by Tor* et al.* [217]. The high capability to remove fluoride at low concentrations with satisfactory adsorption capacity in batch and column adsorption modes and fine reversibility to be regenerated rapidly for four cycles indicate that granular red mud can be used in fluoride adsorption as an upgraded product from powdered red mud adsorbent. Batch experiments indicate that the time to attain equilibrium was 6 h and adsorption was followed the pseudo-second-order kinetic model. Maximum adsorption or removal of fluoride was achieved at pH = 4.7. The adsorption of fluoride by granular red mud in batch systems can be described by the Freundlich isotherm, and the adsorption capacity was 0.851 mg/g.

A new medium, eggshell powder has been developed for fluoride removal from aqueous solutions by Bhaumik* et al.* [218]. Eggshells were collected from local market and washed with double distilled water followed by drying in a hot air oven at 110 °C for overnight. The dried eggshells were ground and sieved well into fractions of 100, 150, 250, 300, 350 μm mesh sizes that were preserved in different sterilized containers for subsequent use as adsorbents. Fluoride adsorption was studied in a batch system where adsorption was found to be pH dependent with maximum removal efficiency at 6.0. The experimental data was more satisfactorily fitted with the Langmuir isotherm model. Batch experiments were performed to study the applicability of the adsorbent by using fluoride contaminated water collected from affected areas. These results indicate that eggshell powder can be used as an effective, low-cost adsorbent to remove fluoride from aqueous solutions as well as groundwater. Similar study conducted by Lunge* et al.* [219]. Low cost composite adsorbent was synthesized using eggshell powder and eggshell membrane. Eggshell composite was synthesized and evaluated for fluoride removal. Synthesis conditions like aluminum loading, calcination temperature, ratio of eggshell membrane and eggshell in eggshell composite was optimized and studied for fluoride adsorption. Eggshell composite proved to be the best adsorbent among all the synthesized adsorbents with excellent adsorption capacity of 37 mg/g at 30 °C. On comparison of the fitness of Langmuir and Freundlcih isotherms, it is evident that Langmuir model fits well as compared to Freundlich, signifying the monolayer adsorption of fluoride on uniform surface. The results of pH analysis reveals that eggshell composite can be used over a wide range of pH = 3–9. Compared to other adsorbents, eggshell composite was found to be much better in terms of fluoride adsorption capacity and cost. Eggshell composite proved to have potential as an economic adsorbent for fluoride removal.

Bleaching powder also known as chlorinated lime (calcium oxychloride) is a white or mostly white powder that mainly consists of calcium hypochloride. It is widely used as a disinfectant for drinking or swimming pool water and also as bleaching agent. Bleaching powder generally has advantageous characteristics as an economical and viable substitute for other adsorbents for fluoride removal from aqueous solution. One of the major advantages of using bleaching powder for fluoride removal over other chemical treatment methods is that, along with being a disinfectant, it also acts as a defluoridation agent. Kagne* et al.* [220] used bleaching powder for fluoride removal and showed that percentage of fluoride removal increased from 28% to 90.6% with an increase in adsorbent dose from 10 g/L to 100 g/L. However, it was noticed that after a dosage of 50 g/L, there was no significant change in the percentage removal of fluoride. The authors also emphasized that optimum pH for maximum fluoride removal was found to be in range of 6–10, which makes it suitable for treating drinking water, especially in rural areas.

Tamarind (*Tamarindus indica*) is an economically important tropical evergreen tree which grows abundantly in the dry tracts of Central and South Indian States. The hard pod shell is removed and discarded when the fruit is ripe, and the fruit is the chief acidulant used in the preparation of foods.* Tamarindus indica* fruit shells were activated by ammonium carbonate and then carbonized. This material with a BET surface area of 473 m^2^/g was used for defluoridation study [221]. The removal of fluoride ions from aqueous solution was highly dependent on the pH of the solution in many cases, as it alters the surface charge on the adsorbents. For pH values from 3.0 to 7.0, the fluoride uptake capacity of ammonium carbonate activated *Tamarindus indica* fruit shells in shaking and stirring experiments increased from 1.84 mg/g to 16.0 mg/g and from 3.12 mg/g to 19.5 mg/g, respectively. Fluoride uptake capacity falls below a pH value of 7.0 and declines above 12.0 with 3.12 mg/g and 5.14 mg/g for shaking and stirring dynamic studies respectively. In both the shaking and stirring dynamic experiments, the increase in the concentration of co-ions increased their inhibiting ability. Sivasankar* et al.* [222] investigated defluoridation capacities of activated and MnO_2_-coated tamarind fruit shell, using batch and column sorption techniques. The fluoride removal capacity of the sorbents was found to be 1990 mg/kg after the contact time of 30 min, at an optimum pH value of 6.5.

Tomar* et al.* [223] examined a novel, cheap, easily available and eco-friendly adsorbent obtained from treated Citrus limonum (lemon) leaf with the aim of fluoride ion removal from aqueous environment. Batch experiments were performed to study the influence of various experimental variables such as pH of aqueous solution (2–8), adsorbent dose (1–10 g/50 mL fluoride solution), contact time (5–145 min), initial fluoride concentration (2–15 mg/L) and the presence of few competing anions on the adsorption of fluoride on C. limonum (lemon) leaf adsorbent. The authors reported that the most efficient fluoride removal was at pH 2, and that the tested adsorbent showed the maximum defluoridation capacity of 70% of 2 mg/L fluoride ion. The experimental data revealed that both the Langmuir and Freundlich isotherm models fitted with the fluoride sorption process but followed a Freundlich isotherm model very well.

Paudyal* et al.* [224] developed a metal loaded orange waste gel for the removal of trace concentration of fluoride from aqueous solution. Various metal loaded adsorbents were prepared from orange waste through chemical modification followed by loading with metal ions to systematically evaluate the adsorption behaviors. The association of various multi-valent metal ions such as Al^3+^, La^3+^, Ce^3+^, Ti^4+^, Sn^4+^ and V^4+^ with fluoride is high because of their high stability with fluoride ions. Ghimire [225] reported that cerium and lanthanum metal ion loaded phosphorylated orange juice residue gel is also highly effective for fluoride ion removal. Thus, the new material produced from orange juice waste can be used for the treatment of fluoride contaminated wastewater.

Native cellulose fibers were surface modified by poly(*N*,*N*-dimethylaminoethyl methacrylate) in order to obtained the anion adsorbent [226]. This adsorbent had high efficiency in removal of F^−^, AsO^2^^−^ and AsO_4_^3^^−^ from aqueous solutions, even at low initial concentrations. Adsorption kinetics showed that the adsorption equilibrium could be reached within 1 min. The pH value influences the adsorption characteristics because of the pH responsibility of native cellulose fibers with poly(*N*,*N*-dimethylaminoethyl methacrylate). Other anions such as Cl^−^, HCO^3^^−^ and SO_4_^2^^−^ hardly affect arsenic adsorption of the adsorbent but will compete with F^−^ and reduce the F^−^ removal proportion.

An Fe(III)-loaded ligand exchange cotton cellulose adsorbent was synthesized for selective adsorption and fluoride removal from drinking water. The cotton cellulose was modified by crosslinking and activation in order to improve the three-dimensional chelating ability of introduced ligands. For the metal of concern, Fe(III) is primarily considered due to its strong affinity toward fluoride anions, being environmentally safe, and low cost [148].

Ganvir and Das [107] investigated possibility of fluoride removal using rice husk ash which was coated with aluminum hydroxide. Rice husk ash was obtained by burning rice/paddy husk which is an abundantly available and is an inexpensive raw material. The adsorption experiments were conducted with initial fluoride concentrations in range of 10–60 mg/L and 0.1 g of adsorbent, while pH value of the initial fluoride solution was adjusted to about 7. All of the experiments were carried out at 27 °C during 1 h. Adsorption study indicates the adsorption capacity of modified rice husk ash to be 15.08 mg/g, while column studies show 9.5 mg/g capacity. Experiments show strong pH-dependence since the maximum fluoride removal was obtained when the pH was kept at 5.0.

Waste seaweed of the *Ulva japonica*, was modified by multivalent metal ions such as Zr(IV) and La(III) after CaCl_2_ cross-linking to produce metal loaded cross-linked seaweed adsorbents [68]. Adsorption characteristic of adsorbents before and after La(III) and Zr(IV) loading were determined using a 0.5 mmol/L fluoride solution at different pH (2–12) at 30 °C. Maximum sorption potential for fluoride was drastically increased after La(III) and Zr(IV) loading, which were evaluated as 0.58 mmol/g and 0.95 mmol/g, respectively. Mechanism of fluoride adsorption was inferred in terms of ligand exchange reaction between hydroxyl ion on co-ordination sphere of the loaded metal ions of metal modified seaweed waste and fluoride ion in aqueous solution, suggesting high possibility of its application for the treatment of fluoride rich water.

Chen* et al.* [227] developed a ceramic-based adsorbent for removal fluoride from aqueous solution. The ceramic adsorbent was the solid phase with a spherical shape and high fluoride removal efficiency and sufficient mechanical strength to retain its physical integrity after long-time adsorption. Ceramic adsorbents were prepared by cost-effective mixture materials consisting of Kanuma mud, which is widespread in Japan, with zeolite, starch, and FeSO_4_·7H_2_O. Batch experiments were performed to study the influence of various experimental parameters such as contact time (0–48 h), initial fluoride concentration (20–100 mg/L), pH (2–12) and the presence of competing anions on the adsorption of fluoride on a ceramic adsorbent. The experimental data revealed that both the Langmuir and Freundlich isotherm models fitted well with the fluoride sorption process. The maximum adsorption capacity of a ceramic adsorbent for fluoride removal was 2.16 mg/g. The optimum fluoride removal was observed between pH ranges of 4.0 and 11.0. Optimum fluoride removal was observed at pH ranges of 4.0–11.0 indicating that the ceramic adsorbent has promising potential utility in practical application. Fluoride adsorption was reduced in the presence of carbonate, phosphate and sulfate and increased slightly in the presence of chloride and nitrate ions. The ceramic adsorbent also showed good regeneration performances when HCl was used as eluent.

The same group of above mentioned authors [228] also reported results of fluoride removal using porous granular ceramic adsorbents containing dispersed aluminum and iron oxides. Adsorbents were synthesized by impregnation with salt solutions followed by precipitation at 600 °C. The loading capacity of these prepared adsorbents for fluoride was 1.79 mg/g at room temperature. The optimum fluoride removal was observed at pH ranges of 4.0–9.0 indicating that the adsorbent has promising potential utility in practical application. Carbonate and phosphate ions showed an extremely negative effect, nitrate ion showed a slightly positive effect while chloride and sulfate ions did not affect the fluoride removal capacity. The adsorption process fitted well with both the Freundlich and Langmuir isotherm models. Kinetic study results indicated that the adsorption process followed a pseudo-second-order kinetic model. Therefore, the porous granular ceramics with mixed aluminum and iron oxides have good potential by promising environmental materials for fluoride removal from aqueous solution.

Chen* et al.* [229] also reported results of batch tests of fluoride removal using surface-modified granular ceramic with Al-Fe complex. Tests were conducted with the aim of determining the effect of the initial fluoride concentration (5–50 mg/L), pH (2–12), and coexisting ions (chloride, nitrate, sulfate, phosphate, calcium and magnesium). Adsorption capacities of synthesized adsorbents were also determined at different temperatures (20, 30 and 50 °C). Results showed that fluoride adsorption onto Al-Fe complex surface-modified granular ceramic was pH-dependent and the maximum removals of fluoride were obtained at pH in range between 5.0 and 8.0 while further increases of pH showed a negative trend. Fluoride adsorption was reduced in the presence of phosphate and sulfate ions and increased slightly in the presence of chloride and nitrate ions. The leaching test shows that no aluminum or ferric ions leached out from the adsorbents. Chen* et al.* [112] also synthesized and tested two representative samples of different iron impregnated granular ceramics for removal of fluoride in aqueous solution. Their study has demonstrated that both the granular ceramics FeSO_4_·7H_2_O and Fe_2_O_3_ can be used for fluoride removal from aqueous solution, while the granular ceramic FeSO_4_·7H_2_O is more effective for fluoride removal than Fe_2_O_3_. Maximum adsorption of fluoride on FeSO_4_·7H_2_O and Fe_2_O_3_ at pH = 7.0 and 4.0 were 94.23% and 60.48%, respectively.

Zhang* et al.* [129] investigated the possibility of fluoride removal using recycled phosphogypsum waste which was prepare in a form of HAP nanoparticles via microwave irradiation technology. Batch fluoride adsorption experiments were conduced to evaluated effect of adsorbent dose, initial fluoride concentration, contact time, pH and temperature. Therefore, fluoride solutions with initial concentrations ranging from 10 mg/L to 50 mg/L and pH value of 2–11 with adsorbent dose from 1 g/L to 10 g/L were used during different contact time within 24 h at 25, 35, 45 and 55 °C. The authors reported that the maximum fluoride adsorption capacities calculated due to Langmuir-Freundlich model were 19.742, 26.108, 36.914 and 40.818 mg/g for 25, 35, 45 and 55 °C at neutral pH, respectively, while higher capacities for fluoride removal were observed at lower pH. The desorption test showed a higher desorption in alkali medium than in acidic and neutral medium, which the authors related to the fact that fluoride ions may be replaced by hydroxyl anions in alkali medium but they also found that, once fluoride are adsorbed over the surface of HAP nanoparticles, it is very difficult to regenerate.

HAP is a calcium phosphate based bioceramic and used in the medical field as it is the main component of the hard tissues of living bodies such as bones, teeth,* etc.* It has been proven that nanoscale materials offer new possibilities to chemists and the surface properties, electronic structure, coordination,* etc.*, would be modified when material dimensions reach the nanoscale. Nano-HAP was hence used as adsorbent material to remove fluoride from water and it has exhibited high performance for fluoride removal. It has been reported that the surface hydroxyl groups are the active site for adsorbent material and the removal of heavy metals by adsorption depends on the surface site. Therefore, the fluoride removal efficiency should be greatly enhanced by increasing the amount of surface hydroxyl groups on the nano-HAP [230]. HAP is a potential material that could be used for the treatment of water contaminated with fluoride ions. The effects of pH (2.6, 4.6, 7.1, 8.9 and 11.5), contact time (7 min–23 h), initial fluoride concentration (2.0–20 mg/L), and adsorbent dose (0.01–0.1 g/25 mL) on the fluoride adsorption by HAP were studied by Jiménez-Reyes* et al.* [231]. Equilibrium was reached after 16 h of contact time and the maximum sorption of fluoride ions was in the pH range between 5 and 7.3. The highest efficiency in the sorption system was determined by using 0.01 g of HAP and 25 mL of solution. The pseudosecond order model described the kinetic sorption processes, and the Freundlich model, the sorption isotherm process. The effect on the sorbent dosage (mass HAP/volume solution) in the sorption system was significant. The higher adsorption capacity of 4.7 mg/g was obtained using 0.01 g of HAP and 25 mL of solution, while with 0.1 g of HAP and 25 mL of solution up to 96% of fluoride was removed. The maximum sorption of fluoride ions was in the pH range between 5 and 7.3, when the insoluble Ca_5_(PO_4_)_3_OH exchanged hydroxyl functional group for fluoride ion.

The adsorption characteristic of cellulose@HAP nanocomposites synthesized in NaOH/thiourea/urea/H_2_O, regarding fluoride removal,* i.e.*, effects of contact time, pH, initial adsorption concentration and coexisting ions, were examined by Yu* et al.* [146]. The adsorption capacity of fluoride increases with the increasing contact time and the adsorption equilibrium is established within 360 min for fluoride adsorption. Since the pH determined the surface charge and the degree of ionization, the authors emphasized pH as one of key parameters, and reported that higher adsorption capacity was noted at low pH value. The increase of adsorbent dose was reported as a beneficial effect on percentage of fluoride removal due to higher number of active adsorption sites. Analysis of the obtained results, due to Langmuir and Freundlich isotherm models, showed high values of correlation coefficients (*R*^2^ > 0.98) of both isotherm models indicating that both of the models could explain the adsorption process of fluoride on cellulose@HAP. The authors reported that the maximum adsorption capacity from the Langmuir model was 4.2 mg/g, and that application of adsorbent in doses above 3 g/L resulted in residual fluoride concentrations of less than WHO norm up to an initial fluoride concentration of 10 mg/L. It was also reported that the coexisting anions have no significant effect on fluoride adsorption by cellulose@HAP nanocomposites.

Mourabet* et al.* [127,128] in two separate studies investigated the fluoride adsorption onto two materials: HAP and Apatitic tricalcium phosphate. The adsorption characteristic of HAP has been characterized regarding the effects of following process parameters: temperature, initial solution pH, adsorbent dose and initial fluoride concentration [127]. The authors reported that obtained data showed that 88.9% of fluoride can be removed from fluoride solution with initial concentration up to 20 mg/L under the optimal conditions (pH = 4.16, temperature of 39 °C, adsorbent dose of 0.28 g). The maximum adsorption capacity calculated due to Langmuir isotherm model was 3.12 mg/g, while the kinetics study indicated that the fluoride adsorption followed second-order kinetics. The effects of above listed parameters on fluoride removal were also examined using Apatitic tricalcium phosphate [128], and up to 82.3% fluoride was removed from aqueous solution with initial fluoride concentration of up to 60 mg/L under optimal conditions of pH = 4, temperature of 40 °C and adsorbent dose of 0.29 g. The maximum adsorption capacity due to Langmuir adsorption isotherm models was calculated to be 13.88, 14.7 and 15.15 mg/g at 25, 30 and 37 °C, respectively. The kinetics study indicated that the adsorption kinetics of fluoride onto Apatitic tricalcium phosphate also followed second-order kinetics well.

The effects of different parameters such as pH (6–9), initial fluoride concentration (1.5–13 mg/L), contact time (0.5–24 h) and co-existing ions (bicarbonate, sulfate, chloride, nitrate) on fluoride adsorption onto magnesium substituted HAP were studied by Garg and Chaudhari [232]. The authors reported that maximum measured fluoride uptake of synthesized magnesium substituted HAP was 2.66 mg/g, emphasizing that, within 30 min, 25.68% of fluoride was removed, while equilibrium was reached in less than 24 h with 47.94% removal. The fluoride removal from aqueous solution using magnesium substituted HAP showed significant dependence of initial fluoride concentration, contact time and pH while the presence of co-existing anions does not affect the defluoridation capacity.

## 4. Conclusions

Fluoride concentration in water depends on several contributing factors such as pH, temperature, total dissolved solids, alkalinity and hardness. Various technologies are currently available to remove fluoride from water, but adsorption processes are generally considered attractive because of their effectiveness, convenience, ease of operation, simplicity of design and for economic and environmental reasons. Performance comparison of different adsorbents is difficult because of inconsistencies in the data, principally due to different experimental conditions (pH, temperature, ionic strength, particle size, initial fluoride concentration, presence of competing ions,* etc.*).

The pH of water is a dominant factor influencing fluoride adsorption. Generally, fluoride adsorption increases from acidic to near neutral pH and then decreases with increase in pH. Another highly important factor influencing fluoride adsorption is the type and concentration of other ions present in treated water,* i.e.*, the adsorbent’s selectivity for fluoride ions since they can occupy adsorbents’ active sites and thereby reduce theoretical adsorption capacity.

Studies on fluoride removal from aqueous solutions using various adsorbents are described and compared here. The results showed that higher adsorption capacities for fluoride ions possess metal oxides and hydroxides and its binary or trimetal combination. Among the oxides and hydroxides, various titanium, iron and aluminum oxides and hydroxides were most frequently tested and showed the highest adsorption capacities over the wide range of pH as well as a high selectivity for fluoride ions. However, due to its high adsorption capacity, activated alumina is still the most used adsorbent for fluoride removal in practice although performance limits regarding pH-dependence are well known.

The biosorbents, chitin and chitosan, are mostly modified and tested due to fluoride removal. The use of chitosan composites and derivatives for fluoride removal from water is of great interest since they are obtained from natural low cost sources. Fluoride adsorption efficiency of biosorbents usually depends of type of multifunctional group and modification that has been conduced with aim to increase adsorption capacity. Most of the tested biosorbents showed good results in bench scale studies but only some of them were tested with real water samples.

Geomaterials have also shown good adsorption performances during water defluoridation. One study’s result shows that geomaterials adsorb fluoride via both precipitation and sorption. Adsorption capacities and defluoridation efficiency of most tested geomaterials and its modifications depend on initial fluoride concentration, pH and contact time and satisfactory results usually were achieved within fluoride initial concentrations up to 10 μg/L, in an acid environment and during the longer contact time (a few hours).

**Table 2:** Comparative evaluation of various adsorbents for fluoride removal. HAP: hydroxyapatite; HFO: hydrous ferric oxide; and TiO_2_: titanium dioxide.

Adsorbent	Concentration range (mg/L)	pH range	Temperature range (°C)	Contact time (min)	Surface area (m^2^/g)	Model used to calculate adsorption capacity	Maximum adsorption capacity (mg/g)	Reference
Activated carbon (rice straw)	5–20	2–10	25–55	60–1440	122.9	Langmuir	18.9	[85]
Activated carbon (*Morringa Indica*)	2–10	2–12	30–50	5–40	-	Langmuir	0.2314	[208]
Activated carbon (*Acacia farnesiana*)	1.5–15	5–8	Ambient	5–70	720	Freundlich	2.622	[209]
Activated carbon (*Pithacelobium dulce*)	1–8	6–9	Ambient	10–120	-	Freundlich	1.9333	[211]
Activated carbon (*Arachis hypogia*)	2–10	3–12	30–60	60–120	2.12	Freundlich	14.79	[82]
Activated carbon (*Cynodon dactylon*)	2–10	Neutral	30–60	15–195	7.3	Langmuir	4.755	[83]
Activated carbon (*Anacardium occidentale*)	2–10	3–12	30–60	60–210	-	Langmuir	1.95	[84]
Activated carbon (pecan nut shells)	5–40	Neutral	30	2160	17	Langmuir	2.3	[86]
Alginate (*Ulva japonica*)	21–252	2–11	30	1140	-	Langmuir	39.9	[182]
Aluminum modified zeolitic tuff	0.5–10	5.5–8	Ambient	50–4320	139.22	Langmuir-Freundlich	10.25	[197]
Aluminum (hydr)oxide coated pumice	5	3–11	20	0–4800	1.5	Langmuir	7.87	[109]
Alginate entrapped Fe(III)-Zr(IV) binary mixed oxide	10	2–12	10–50	5–300	74.61	Langmuir	0.981	[119]
Apatitic tricalcium phosphate	30–60	4–11	20–40	90	-	Langmuir	15.15	[128]
Cerium dispersed in carbon	2.8–8.3	5.5–9	25–65	5–60	685	Langmuir	209	[122]
Calcined Mg/Fe layered double hydroxide	5–50	2.5–11	25	0–600	145.3	Langmuir	50.91	[124]
Calcium chloride modified natural zeolite	25–100	4–9	25–45	5–1200	-	Langmuir	1.766	[198]
Cellulose@HAP nanocomposites	5–10	4-9	25	5–700	76.257	Freundlich	2.76	[146]
CeO_2_-ZrO_2_ nanocages	5–40	2–8	25–55	0–1440	29.61	Langmuir	175	[135]
Fe–Al–Ce nano-adsorbent	42	6.5–7.5	Ambient	2160	-	Langmuir	2.77	[136]
Fe–Al–Ce hydroxide	10–250	7	25	1440	56.4	Langmuir	51.3	[137]
Fe-Ti oxide nano-adsorbent	50	6.9	Ambient	720	-	Langmuir	47.0	[143]
Graphene	5–40	3.6–10.2	0–50	1–110	3.08	Langmuir	48.31	[79]
Hydrogen peroxide modified pumice	5–20	2–10	10–50	0–210	53.11	Freundlich	11.765	[194]
Hydrous bismuth oxides	10–35	4–12	20–40	60–360	76.042	Langmuir	1.93	[121]
Hydrous zirconium oxide	2–120	3–10	25	5–700	134	Freundlich	124	[130]
HFO doped alginate beads	5–10	3.5–9	20–40	0–3600	25.80	Langmuir	8.90	[116]
HAP nanoparticles	10–50	2–11	25–55	60–1440	-	Langmuir-Freundlich	40.818	[129]
Mg-doped nano ferrihydrite	10–150	1–10	20–45	30–480	248.6	Langmuir	64	[141]
Meixnerite (calcined)	12.4–248	-	20	30–1800	-	Langmuir	56.8	[150]
Nitrate containing ZnCr layered double hydroxides	0–100	3–10	Ambient	0–1440	12	Langmuir	31	[134]
Pumice	2–7	4–9	Ambient	0–180	-	Langmuir	0.31	[101]
Siderite (modified)	2–25	2–12	15–45	10–780	79.52	Langmuir	5.460	[104]
Sm(III)-loaded orange waste	10–240	1–8	30	0–1440	-	Langmuir	1.22	[184]
Stilbite zeolite modified with Fe(III)	5–40	3–11	Ambient	15–180	-	Langmuir	2.31	[156]
Surfactant-modified pumice	1.5–20	3–10	20–30	5–1140	11.79	Langmuir	41	[108]
Sulfate-doped Fe_3_O_4_/Al_2_O_3_ nanoparticles	2–160	2–12	Ambient	5–540	63.37	Langmuir	70.4	[161]
TiO_2_	2–20	2–11	Ambient	5–1140	-	Langmuir	0.2703	[160]
Zeolite (Ethiopia)	22.1	8.5	Ambient	1200	-	-	0.47	[157]
Zirconium(IV)-ethylenediamine hybrid material	2–50	2–12	10–50	2–30	196.5	Dubinin-Radushkevich	37.03	[133]
Zirconium-iron oxide	10–150	3–11	25	5–840	95.5	Freundlich	9.80	[126]
Zirconium phosphate	1–10	2–12	10–50	2–60	129	Langmuir	4.268	[131]

The most commonly used adsorbents belong to a group of carbonaceous materials and activated carbons, respectively. Fluoride adsorption onto carbonaceous materials depends on base material, activation process, valence of metal ions used for its modification, and pore size distribution since adsorption occurs mainly in the pores of the material. The advantages of carbonaceous materials, especially of modified activated carbons, are high adsorption capacities and partially good regeneration properties, but their maximum adsorption performances are usually strongly pH dependent.

Different industrial products and by-products have been tested for fluoride removal due to its ecological and reuse aspects and the fact that those materials are available in significant quantities and at low prices. Although authors usually report good fluoride uptake and high adsorption capacities of those adsorbents, most of them were strongly pH-dependent and authors often report problems with difficult adsorbent regeneration.

Final comparative evaluation of various adsorbent discussed in this review paper are presented in Table 2.
